# Deep Learning Methods in Medical Image-Based Hepatocellular Carcinoma Diagnosis: A Systematic Review and Meta-Analysis

**DOI:** 10.3390/cancers15235701

**Published:** 2023-12-03

**Authors:** Qiuxia Wei, Nengren Tan, Shiyu Xiong, Wanrong Luo, Haiying Xia, Baoming Luo

**Affiliations:** 1 Department of Ultrasound, Sun Yat-sen Memorial Hospital, Sun Yat-sen University, 107 West Yanjiang Road, Guangzhou 510120, China; weiqx5@mail2.sysu.edu.cn (Q.W.); xiongshy5@mail3.sysu.edu.cn (S.X.); luowr8@mail2.sysu.edu.cn (W.L.); 2 Guangdong Provincial Key Laboratory of Malignant Tumor Epigenetics and Gene Regulation, Sun Yat-sen Memorial Hospital, Sun Yat-sen University, 107 West Yanjiang Road, Guangzhou 510120, China; 3 School of Electronic and Information Engineering, Guangxi Normal University, 15 Qixing District, Guilin 541004, China; tannengren@stu.gxnu.edu.cn

**Keywords:** deep learning methods, medical Image, hepatocellular carcinoma, diagnosis

## Abstract

**Simple Summary:**

In this study, after conducting a comprehensive review of 1356 papers that evaluated the diagnostic performance of deep learning (DL) methods based on medical images for hepatocellular carcinoma (HCC), the findings showed a pooled sensitivity of 89% (95% CI: 87–91), a specificity of 90% (95% CI: 87–92), and an AUC of 0.95 (95% CI: 0.93–0.97). In addition, both the DL methods and human clinicians demonstrated similar levels of performance in HCC detection, with receiver operating characteristic curve (ROC) values of 0.97 (95% CI: 0.95–0.98) for both groups, indicating no discernible difference. Although the heterogeneity was obvious, the utilization of DL methods for diagnosing HCC through medical images has shown promising outcomes.

**Abstract:**

(1) Background: The aim of our research was to systematically review papers specifically focused on the hepatocellular carcinoma (HCC) diagnostic performance of DL methods based on medical images. (2) Materials: To identify related studies, a comprehensive search was conducted in prominent databases, including Embase, IEEE, PubMed, Web of Science, and the Cochrane Library. The search was limited to studies published before 3 July 2023. The inclusion criteria consisted of studies that either developed or utilized DL methods to diagnose HCC using medical images. To extract data, binary information on diagnostic accuracy was collected to determine the outcomes of interest, namely, the sensitivity, specificity, and area under the curve (AUC). (3) Results: Among the forty-eight initially identified eligible studies, thirty studies were included in the meta-analysis. The pooled sensitivity was 89% (95% CI: 87–91), the specificity was 90% (95% CI: 87–92), and the AUC was 0.95 (95% CI: 0.93–0.97). Analyses of subgroups based on medical image methods (contrast-enhanced and non-contrast-enhanced images), imaging modalities (ultrasound, magnetic resonance imaging, and computed tomography), and comparisons between DL methods and clinicians consistently showed the acceptable diagnostic performance of DL models. The publication bias and high heterogeneity observed between studies and subgroups can potentially result in an overestimation of the diagnostic accuracy of DL methods in medical imaging. (4) Conclusions: To improve future studies, it would be advantageous to establish more rigorous reporting standards that specifically address the challenges associated with DL research in this particular field.

## 1. Introduction

Liver cancer, also known as HCC, is a prevalent and deadly form of cancer, ranking as the sixth most common type worldwide and the third leading cause of mortality [1]. Early-stage HCC often lacks noticeable symptoms, which can lead to delayed diagnosis as the cancer progresses. Symptoms, such as fatigue, weight loss, or abdominal discomfort, can be nonspecific and resemble other liver diseases, such as cirrhosis and hepatitis. This similarity poses challenges in differentiating and promptly diagnosing HCC [2,3]. HCC is characterized by tumor heterogeneity, which can impact the accuracy of tissue sampling and biopsy results, further complicating diagnosis confirmation [4]. Therefore, accurate and reliable technologies are crucial for the effective early detection of HCC.

Medical imaging is essential in clinical practice for diagnosis, staging, and treatment planning. Modalities such as ultrasound (US), magnetic resonance imaging (MRI), and computed tomography (CT) are noninvasive and offer valuable tumor images, reducing patient discomfort and risks compared to invasive procedures like biopsies [5,6,7]. These techniques provide detailed anatomical and pathological information, aiding in determining tumor characteristics such as size, location, and malignancy. However, the interpretation of medical images still relies on the subjective judgment and experience of healthcare professionals. There can be variability in diagnostic results among different doctors, introducing subjectivity [8,9]. Given the variation in expertise, achieving accurate and timely diagnoses based on medical images remains challenging.

DL is a machine learning technique that includes multiple model architectures and can solve various types of machine learning problems. Common DL methods are based on convolutional neural networks, recurrent neural networks, long short-term memory networks, generative adversarial networks, etc. DL methods have shown promising results in the automatic detection of medical images, enabling automatic diagnosis and classification of diseases by analyzing and identifying features and lesions [10,11]. Compared to manual analysis, DL methods offer faster processing and improved efficiency, reducing the burden on doctors. DL methods typically consist of several steps: building a DL model, collecting and processing data, setting model parameters, completing model training, and evaluating and tuning the model. In addition, the dataset used by DL methods can usually be divided into the training set, the validation set, and the test set. The data can be augmented, cropped, and processed by other enhancement methods. DL methods use multi-level feature extraction networks to simulate and learn complex features of data. They learn complex visual patterns and features from much medical image data, enhancing diagnostic accuracy. DL methods outperform traditional approaches by capturing more diagnostically significant subtle features, aiding in the accurate assessment of pathological changes [12,13]. Additionally, DL models analyze large-scale medical image data, revealing hidden patterns and correlations, thus improving the understanding of disease mechanisms, variations, progression, and prognosis [14,15]. However, there is currently a lack of comprehensive evidence on the use of DL-based methods for HCC detection. Accordingly, this study aimed to provide a systematic review and meta-analysis of published data to evaluate the diagnostic performance of DL methods based on medical images in detecting HCC.

## 2. Methods

### 2.1. Protocol Registration and Study Design

We registered our study protocol in PROSPERO with the number CRD42023442527. The study followed the guidelines outlined in the Preferred Reporting Items for Systematic Reviews and Meta-Analyses (PRISMA) to ensure comprehensive and transparent reporting [16] and Assessing the Methodological Quality of Systematic Reviews (AMSTAR) guidelines [17]. Informed consent from all subjects (patients) was not required because our data came from the open database.

### 2.2. Search Strategy and Eligibility Criteria

A systematic search was conducted with several databases, including Embase, IEEE, PubMed, Web of Science, and the Cochrane Library. The search aimed to identify studies published from the inception of the database to July 3, 2023 that focused on the development of DL methods for diagnosing HCC based on medical images. Supplementary Note SI summarized the search terms and search strategy used in each database. No limitations were imposed regarding publication types, regions, or language. However, conference abstracts, scientific reports, letters, and narrative reviews were excluded. A team of clinicians and investigators collaboratively developed a comprehensive search strategy for each database.

Two investigators assessed eligibility by screening titles, abstracts, and relevant citations. Discrepancies were resolved through discussion with an additional contributor. Inclusion criteria consisted of studies reporting DL models’ diagnostic performance in early HCC detection using medical images. Studies that reported diagnostic results such as sensitivity and specificity or detailed information on 2 × 2 contingency tables were considered eligible for inclusion. The use of DL models was not limited by participant characteristics, imaging modality, or intended setting.

### 2.3. Data Extraction

The study characteristics and diagnostic yield data were independently extracted by two investigators using a standardized data extraction sheet. Uncertainties were resolved through discussions with a third researcher. With a meticulous approach, we diligently extracted the diagnostic accuracy data and precisely organized it into contingency tables, including the number of true positives (TPs), false positives (FPs), true negatives (TNs), and false negatives (FNs). 

### 2.4. Study Quality Assessment

Three researchers utilized the QUADAS-2 tool to assess both the risk of bias and concerns about the suitability of the included studies [18]. This tool was specifically chosen to aid in the evaluation process.

### 2.5. Statistical Analysis

Hierarchical SROC curves assessed the DL methods’ diagnostic performance, presenting averaged estimates of sensitivity, specificity, AUC, and 95% CI with prediction regions. A meta-analysis using contingency tables identified the most accurate DL methods across studies with multiple methods. Heterogeneity was evaluated with the *I^2^* statistic, exploring potential sources through subgroup meta-analyses and regression analyses. The random-effects model accounted for the substantial heterogeneity. Publication bias was assessed visually with funnel plots.

In the process of collating the data, we found that DL methods combined with contrast-enhanced images had higher diagnostic accuracy than non-contrast-enhanced images. The following subanalyses were further conducted: (a) Based on the medical image method, the DL methods were divided into two categories: contrast-enhanced and non-contrast-enhanced images. Image enhancement methods used contrast media, and non-contrast-enhanced images did not use contrast media. (b) The DL methods were categorized by their respective imaging modalities, including CT, US, and MRI. (c) The DL methods were classified as internal or external methods depending on the type of validation. Internal validation was conducted using internal data for validation. External validation was conducted using external data for validation. (d) The DL methods were assessed and compared with human clinicians based on aggregated performance measures using the same datasets. Meta-analyses were conducted if at least three original studies were available. We harnessed the mighty STATA (version 17.0) to dissect our data with precision. Our threshold for significance was set at *p* < 0.05, and we employed a robust two-sided approach to determine statistical significance for all tests. 

## 3. Result

### 3.1. Study Selection and Characteristics

**Figure 1:**
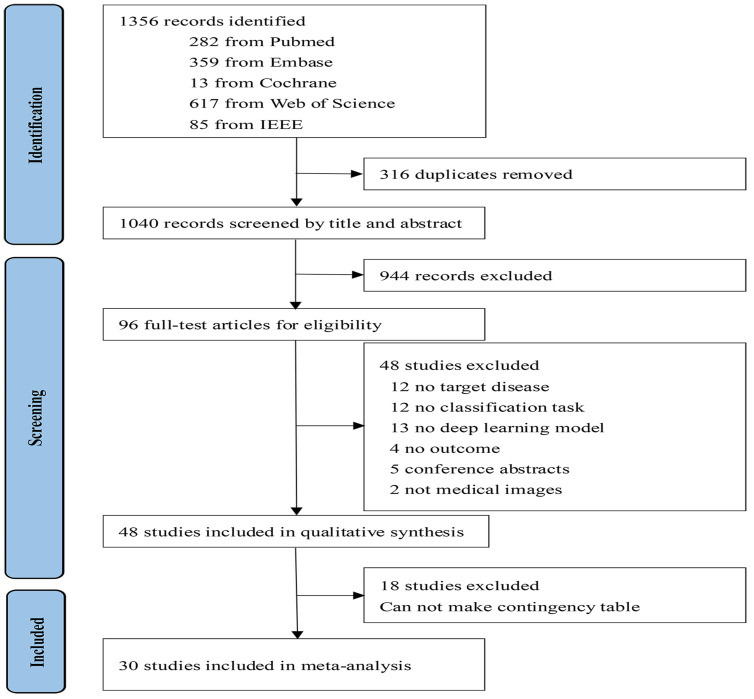
Flowchart of study selection.

As shown in Figure 1, after removing 316 duplicates, our initial search yielded 1356 records, of which 1040 underwent screening. From the title and abstract screening, 944 studies were excluded; therefore, there were 96 for further full-text screening. Ultimately, 48 articles [19,20,21,22,23,24,25,26,27,28,29,30,31,32,33,34,35,36,37,38,39,40,41,42,43,44,45,46,47,48,49,50,51,52,53,54,55,56,57,58,59,60,61,62,63,64,65,66] were considered appropriate in our review, with 30 [19,20,21,22,23,24,25,26,27,28,29,30,31,32,33,34,35,36,37,38,39,40,41,42,43,44,45,46,47,48] offering data for further meta-analysis. Among these studies, 45 utilized retrospective data, 1 employed prospective data, and 2 sourced data from open-access sources. Out of the identified studies, 5 utilized out-of-sample datasets for external validation. Five studies compared the performance of the DL methods to that of clinicians utilizing the identical dataset. Medical imaging modalities were classified into the following categories: MRI (*n* = 10), US (*n* = 7), and CT (*n* = 13). Table 1 and Table 2 showed the characteristics of the included study.

### 3.2. Overall Performance of the DL Methods

**Figure 2:**
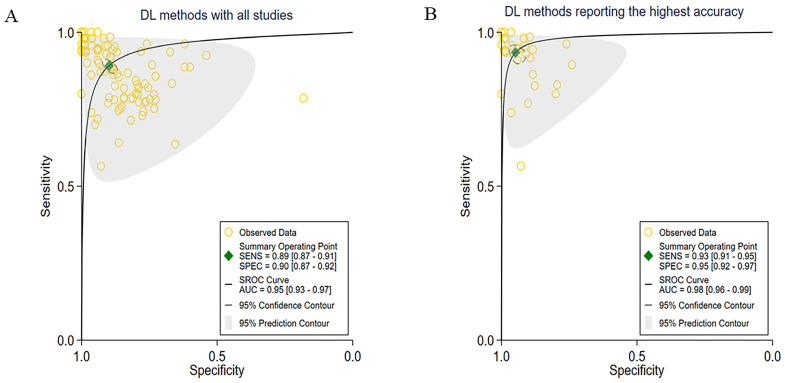
A comprehensive evaluation of DL methods. (**A**) Receiver operator characteristic (ROC) curves of all studies included in the meta-analysis (102 tables of 30 studies), and (**B**) ROC curves of studies reporting the highest accuracy (30 tables of 30 studies).

Out of the 48 studies included, 30 offered enough data for creating contingency tables and calculating diagnostic yields, making them eligible for the meta-analysis. The meta-analysis included 102 contingency tables, as shown in Figure 2A, with a pooled sensitivity of 89% (95% CI: 87–91) and specificity of 90% (95% CI: 87–92) across all the DL methods. The AUC was determined to be 0.95 (95% CI: 0.93–0.97).

Since most studies investigated multiple DL methods and reported their diagnostic performance, we chose to report the highest accuracy achieved by various DL methods across the included studies, which resulted in 30 contingency tables. As we combined their findings, we discovered a pooled sensitivity of 93% (95% CI: 91–95) and a specificity of 95% (95% CI: 92–97). The AUC was calculated to be 0.98 (95% CI: 0.96–0.99). More detailed information was shown in Figure 2B.

### 3.3. Subgroup Meta-Analyses

**Figure 3:**
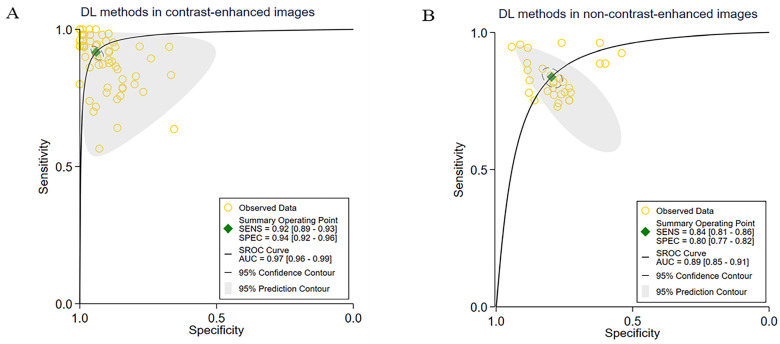
Pooled performance of DL methods using different medical image methods. (**A**) ROC curves of studies with contrast-enhanced images (65 tables of 23 studies), (**B**) ROC curves of studies with non-contrast-enhanced images (30 tables of 6 studies).

Among the studies included in the analyses, 23 studies focused on contrast-enhanced images, resulting in a total of 65 contingency tables. The pooled sensitivity for these studies was 92% (95% CI: 89–93), while the pooled specificity was 94% (95% CI: 92–96). Additionally, the AUC was 0.97 (95% CI: 0.96–0.99). More detailed information is shown in Figure 3A. Furthermore, 6 studies did not investigate image contrast enhancement, contributing a total of 30 contingency tables. The pooled sensitivity of these studies was 84% (95% CI: 81–86), the pooled specificity was 80% (95% CI: 77–82), and the AUC value was 0.89 (95% CI: 0.85–0.91). More details regarding these studies could be found in Figure 3B. 

**Figure 4:**
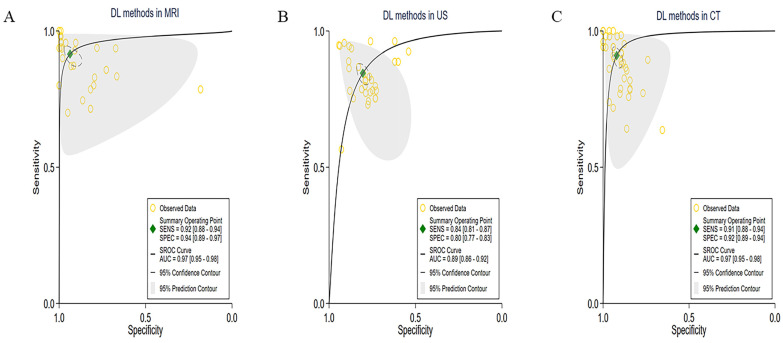
Pooled performance of DL methods using different imaging modalities. (**A**) ROC curves of studies using MRI (29 tables of 10 studies), (**B**) ROC curves of studies using US (32 tables of 7 studies), and (**C**) presented ROC curves of studies using CT (41 tables of 13 studies).

Among the studies included in the analyses, 10 studies investigated MRI data, which resulted in a total of 29 contingency tables. The pooled sensitivity of these studies was 92% (95% CI: 88–94), and the pooled specificity was 94% (95% CI: 87–97). Furthermore, the AUC was 0.97 (95% CI: 0.95–0.98). More detailed information about these studies is shown in Figure 4A. In addition, 7 ultrasound studies were analyzed, contributing a total of 32 contingency tables. The pooled sensitivity of these studies was 84% (95% CI: 81–87), the pooled specificity was 80% (95% CI: 77–83), and the AUC value was 0.89 (95% CI: 0.86–0.92). More detailed information regarding these studies is provided in Figure 4B. Furthermore, 13 studies applied CT data, resulting in a total of 41 contingency tables. The pooled sensitivity of these studies was 91% (95% CI: 88–94), the pooled specificity was 92% (95% CI: 89–94), and the AUC value was 0.97 (95% CI: 0.95–0.98). More detailed information about these studies is presented in Figure 4C.

**Figure 5:**
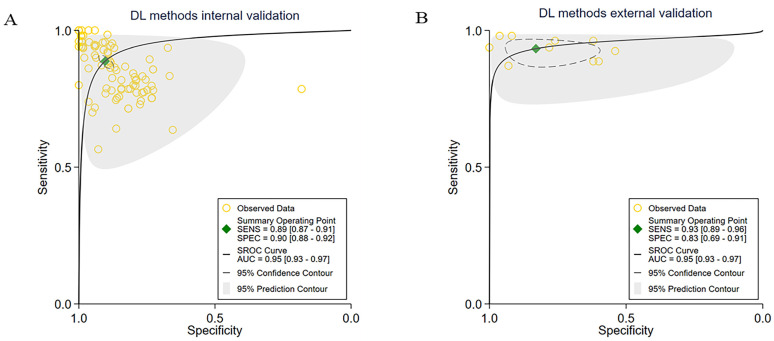
Pooled performance of DL methods using different validation types. (**A**) ROC curves of studies with internal validation (92 tables of 29 studies), (**B**) ROC curves of studies with external validation (10 tables of 5 studies).

The meta-analysis included 29 studies that used within-sample datasets, comprising a total of 92 contingency tables. For these studies, the pooled sensitivity and specificity were 89% (95% CI: 87–91) and 90% (95% CI: 88–92), respectively. The AUC was determined to be 0.95 (95% CI: 0.93–0.97), as illustrated in Figure 5A. External validation was performed in only 5 studies, contributing 10 contingency tables. The pooled sensitivity and specificity for these studies were 93% (95% CI: 89–96) and 83% (95% CI: 69–91), respectively. The AUC was calculated as 0.95 (95% CI: 0.93–0.97), as shown in Figure 5B.

**Figure 6:**
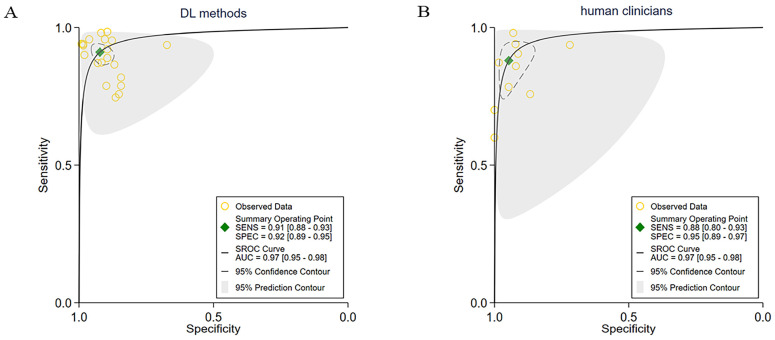
Pooled performance of DL methods versus human clinicians using the same sample. (**A**) ROC curves of studies using DL methods (20 tables of 6 studies), and (**B**) ROC curves of studies using human clinicians (10 tables of 6 studies).

Among the 30 included studies, 6 directly compared the diagnostic performance of the DL methods with human clinicians using the same dataset. These studies consisted of 20 contingency tables for the DL methods and 10 contingency tables for the human clinicians. The pooled sensitivity for the DL methods was 91% (95% CI: 88–93), while the human clinicians had a pooled sensitivity of 88% (95% CI: 80–93). The pooled specificity for the DL methods was 92% (95% CI: 89–95), compared to 95% (95% CI: 89–97) for the human clinicians. Both the DL methods and the human clinicians exhibited an AUC value of 0.97 (95% CI: 0.95–0.98), as depicted in Figure 6A,B.

### 3.4. Heterogeneity Analysis

The meta-analysis of 30 studies indicated that the DL methods were beneficial in diagnosing HCC from medical imaging based on the random-effects model. However, the sensitivity showed an *I^2^* value of 99.83% (*p* < 0.01) and the specificity had an *I^2^* value of 99.84% (*p* < 0.05), indicating high heterogeneity. Supplementary Figure S4 presents more details on these results. The detailed results of the subgroup (Supplementary Figures S5–S7) and meta-regression analyses (Supplementary Table S1) explored the potential sources of between-study heterogeneity. Apart from the imaging modality, both medical image methods and validation types demonstrated statistically significant differences, corroborating the findings from the subgroup. To evaluate publication bias, a funnel plot was generated. The result showed that this study had obvious publication bias (*p* < 0.05). More detailed information was presented in Supplementary Figure S3.

### 3.5. Quality Assessment

QUADAS-2 was used to assess the quality of the included studies, and the results were summarized in Supplementary Figure S1. Supplementary Figure S2 present a detailed evaluation of each item related to the risk of bias and applicability concerns. For patient selections (*n* = 30) and reference standards (*n* = 22), over half of the studies demonstrated a high or unclear risk of bias. This was mainly due to a lack of clarity in describing the included patients, such as previous testing, presentation, setting, intended use of the index test, and insufficient external evaluation.

## 4. Discussion

Through this study, we assessed the diagnostic effectiveness of DL methods for HCC detection based on medical images. When averaging the results across the studies, the pooled sensitivity, specificity, and AUC were found to be 89%, 90%, and 0.95, respectively. When determining the highest accuracy of each DL method among the included studies, we found that the DL methods demonstrated superior performance in terms of sensitivity (93%), specificity (95%), and AUC (0.98). In subgroup analysis, to begin with, we found that DL methods combined with contrast-enhanced images had higher diagnostic accuracy than non-contrast-enhanced images. The reason may be that the image enhancements have a higher resolution ratio so that tiny lesions are displayed more clearly, which is more conducive to the diagnosis of cancer. Furthermore, MRI, US, and CT are the main imaging techniques for the diagnosis of HCC. The selection of imaging techniques for HCC diagnosis depends on several factors, including the patient’s condition, availability of medical resources, and specific circumstances. Typically, doctors choose the most suitable imaging examination based on each patient’s needs and the characteristics of their condition. Moreover, using an internal dataset may overstate diagnostic value since homogeneity is produced, but external validation through out-of-sample data can offer insights into subgroups and variations among different ethnic groups. However, the presence of high heterogeneity and variance between studies results in considerable uncertainty surrounding the estimates of diagnostic accuracy in this meta-analysis.

A systematic search for relevant articles resulted in the identification of four systematic reviews or meta-analyses that explored the significant role of artificial intelligence (AI) and medical images in HCC diagnosis. However, these reviews considered diverse domains, making direct comparisons with this research challenging. Chou et al. discovered that image-based diagnosis of HCC had a sensitivity of 84% and specificity of 99%, highlighting its importance. However, they did not explore AI methods [67]. In our research, with the assistance of the DL method, the effectiveness of medical image diagnosis of HCC was further improved. Lai showed that AI methods outperformed traditional systems in predicting HCC treatment outcomes, but their review lacked sufficient data for a meta-analysis [68]. The meta-analysis we conducted can reduce the differences caused by random errors and increase the efficiency of the tests. Martinino et al. observed that as the number of studies and images increased, AI methods became more effective in diagnosing HCC, but the review did not differentiate between machine learning and DL methods [69]. By applying DL methods to assist in the diagnosis of HCC, we can automatically learn patterns and features from the data to achieve more accurate predictions and decision-making. Zhang et al.’s meta-analysis revealed that DL methods excel in predicting microvascular invasion, demonstrating superior accuracy, methodology, and cost-effectiveness. However, HCC classification was not investigated in their study [70]. Therefore, our research perfectly filled this gap. 

Our research showed that DL methods are a powerful tool in diagnosing HCC, and we summarize our results as follows. First, the DL methods can extract intricate patterns and features based on medical images, enabling accurate identification of early-stage liver tumors that may be challenging for human experts to detect [71]. This early detection had great significance in improving treatment outcomes and increasing patient survival rates [72]. Second, DL models can process and analyze much imaging data in a relatively short time, facilitating faster and more efficient diagnoses [73,74]. With this improved speed and efficiency, patients can promptly receive their diagnosis, allowing for expedited treatment planning and intervention. Moreover, the DL methods can learn based on large datasets of medical images to continuously improve their accuracy and performance [75,76]. This adaptive learning capability enables the methods to remain up-to-date with the latest medical knowledge and advancements in HCC detection, ensuring the most accurate and reliable diagnoses. Another advantage of DL in HCC diagnosis is its potential for reducing human subjectivity and variability. By relying on objective image analysis, the DL methods can provide consistent and standardized evaluations, leading to more reliable and reproducible diagnoses [77,78]. This consistency is especially valuable in cases in which doctors’ opinions may differ, as the methods can provide an additional objective perspective. Furthermore, DL models can integrate multiple imaging modalities, such as CT scans, MRI, and ultrasound, to provide a more comprehensive and holistic assessment than other methods [79,80]. By fusing information from various sources, these models can enhance the accuracy and reliability of HCC diagnosis and help guide treatment decisions. Lastly, with the development of the social economy, the quality of datasets obtained in HCC research is constantly improving, the data is increasing, and the diversity of data is constantly enriched. Meanwhile, the rapid development of DL methods has continuously made breakthroughs in algorithm innovation. The advanced performance of deep learning methods is based on mass of data for training because the accurate features will be obvious for the training effect on the mode to improve the generalization ability of the model. If the data is insufficient, the training of deep learning models is fatal, resulting in the model training appearing to be overfitting. Of course, this problem can be solved by data augmentation, but the generalization ability of the model may not be improved. We included the latest articles that published up until 2023, and the number of training sets can be up to hundreds of thousands; thus, our article with higher indicators (AUC and ROC) compared to similar articles is acceptable. Overall, DL has considerable advantages in HCC diagnosis, including improved early detection, faster processing times, continuous learning and improvement, reduced subjectivity, and more comprehensive evaluations. The integration of DL methods into clinical practice can significantly enhance patient care and outcomes in HCC.

Our study had some limitations. First, there was evident heterogeneity in our study. Despite subgroup and meta-regression analyses being carried out, the heterogeneity could not be completely eliminated. Second, due to limited data, we could not perform subgroup analysis based on tumor size and location. Third, the included studies were almost entirely retrospective, and potential confounding variables and confounding bias may limit the internal validity of retrospective studies. Research on DL methods based on medical images for HCC diagnosis should be improved in terms of study design.

## 5. Conclusions

In conclusion, the DL methods based on medical images for detecting HCC were found to be highly accurate, although the heterogeneity is obvious. Furthermore, the sensitivity of the DL methods significantly improved when utilizing contrast-enhanced imaging techniques.

## Figures and Tables

**Table 1:** Study design and basic demographics.

First Author and Year	Participants Inclusion Criteria	Participants Exclusion Criteria	Reference Standard	Patients (Number)
Liu et al. 2023 [41]	Patients who (1) underwent a preoperative MRI examination; (2) had no history of treatment for hepatic tumor prior to the study; (3) pathologically confirmed HCC or MF-ICC	Patients with (1) image quality were insufficient for further analysis; (2) T2WI-MRI was incomplete	Histopathology	112
Murtada et al. 2023 [42]	NA	NA	NA	59
Abhishek et al. 2023 [43]	Patients who had abdominal CT scans within three months of operation with a routine clinical imaging protocol of contrast-enhanced portal venous phase CT	Patients who had (1) no contrast-enhanced CT scans; (2) metal artifacts infiltrating the tumor on CT imaging; (3) prior ablation, embolization, resection, or transplantation, as these prior treatments would alter the appearance of the tumors on imaging and compromise the quantitative image analysis; (4) tumors that were ruptured; (5) tumors with a diffuse infiltrative pattern (as tumor borders were challenging to determine for analysis)	Histopathology	814
Anisha et al. 2023 [44]	NA	NA	NA	320
Huang et al. 2023 [45]	Patients with pathologically confirmed HCC or ICC who underwent hepatectomy	Patients with pathologically confirmed HCC or ICC who underwent hepatectomy	Histopathology	1042
Zhang et al. 2023 [47]	NA	NA	NA	317
Mitrea et al. 2023 [46]	NA	NA	Histopathology	296
Wang et al. 2023 [48]	Patients who (1) were at least 18 years old; (2) had clear CT image with lesion location being analyzed easily; (3) had no other genetic history in the family	Patients who (1) take related prohibited drugs before CT image acquisition; (2) during hospital examination, the patient had a severe malignant tumor and other systemic diseases	NA	102
Ling et al. 2022 [38]	NA	NA	Histopathology	479
Cao et al. 2022 [39]	Patients who (1) were diagnosed with HCC or HCH based on liver biopsy or clinical findings; (2) had no contraindications to contrast medium and had undergone upper abdominal contrast-enhanced CT scans	NA	Histopathology	50
Zhang et al. 2022 [40]	Patients who were pathologically confirmed as HCC or FNH after surgical resection	Patients who (1) have complicated clinical conditions such as pregnancy and taking medication for collagen diseases; (2) received additional treatment before examination such as chemotherapy, radiofrequency ablation (RFA), or transcatheter arterial chemoembolization (TACE)	Histopathology	407
Gao et al. 2021 [30]	Patients who were (1) pathologically confirmed with one of the following malignant hepatic tumors: HCC, ICC, and metastasis; (2) with preoperative multi-phase contrast-enhanced CT available	Patients (1) who were ≤18 years old; (2) who had a prior liver resection or transplantation; (3) whose interval between the pathologic examination and the preoperative CT > 100 days; (4) whose image quality was poor	Histopathology	723
Oestmann et al. 2021 [34]	Patients had histopathological diagnosis and were older than 18 years	NA	Histopathology	118
Wang et al. 2021 [31]	The HCC group consisted of patients not only treated by surgical resection but also treated by intervention, radiofrequency ablation, cryoablation, microwave therapy, or any other invasive treatment therapy. Both solitary and multiple HCC tumor nodules were enrolled. Patients diagnosed with malignant lesions other than HCC such as hemangioendothelioma, sarcoma, intrahepatic cholangiocarcinoma, and metastatic tumor were included in the control group. Patients diagnosed with benign lesions such as leiomyolipoma, hemangioma, cyst, abscess, adenoma, and focal nodular hyperplasia were also included in the control group	NA	Histopathology	9741
Wang et al. 2021 [33]	Patients who (1) had liver surgical resection or biopsy in the period between 2006 and 2019; (2) were diagnosed with HCC, ICC, or secondary metastasis lesion	Patients who (1) lost images or stored images in other hospitals; (2) only had other types of scans	Histopathology	400
Wang et al. 2021 [37]	Patients who (1) didn’t have MRI inspection; (2) had one of the following common FLLs, including liver cyst, HEM, HEP, FNH, HCC, ICC, and MET; (3) had up to one imaging study per patient and up to six lesions being used in each study	Patients (1) with MRI studies of insufficient image quality; (2) had received treatment related to the lesion before MRI inspection; and (3) had diffuse lesions for which the boundary could not be delineated or malignancies involving the portal vein, hepatic vein, or adjacent organs	NA	445
Zhou et al. 2021 [32]	Patients with definite pathological results of non-cystic FLL were registered	Patients with (1) benign lesions; (2) without cirrhosis; (3) with previous treatment; (4) without US images; (5) lesion size < 1.0 cm; (6) unsatisfied US image quality	Histopathology	172
Shi et al. 2020 [27]	Patients (age ≥ 18 years) with FLLs other than cysts underwent four-phase CT exams	Patients with (1) lesions that could not be reliably classified by the best available reference standard as HCC or non-HCC; (2) lesion sizes below 1 cm; (3) CT exams with fewer than four phases or with severe image artifacts; (4) previous transcatheter arterial chemoembolization or other previous locoregional therapy; (5) loss to follow-up (*n* = 13)	Histopathology, clinical diagnosis, and follow-up	915
Zhen et al. 2020 [25]	Patients with (1) liver tumors and (2) enhanced MRI inspection	Patients with (1) treatment related to the lesion before MRI inspection, including surgery, transcatheter arterial chemoembolization (TACE), radiofrequency ablation, chemotherapy, radiotherapy, targeted drug therapy, etc.; (2) inflammatory lesions; (3) a clinically diagnosed malignancy (without pathology confirmed); (4) any missing important medical records or laboratory results of the malignancy individuals; and (5) unqualified image quality	Histopathology, clinical diagnosis, and follow-up	1411
Kim et al. 2020 [28]	Patients who were diagnosed as HCC after surgical resection	Patients with (1) severe motion artifacts; (2) missing images; (3) low image quality; (4) absence of preoperative MR images	Histopathology	549
Cao et al. 2020 [29]	Patients with (1) the images of a four-phase DCE-CT examination; (2) FLLs confirmed by histopathological evaluation; (3) a diagnosis based on a combination of clinical and radiological findings with follow-up were collected for further screening	Patients with (1) lesions larger than 10 cm; (2) images with prominent artifacts; (3) prior local-regional therapy prior to the CT examination.	Histopathology, clinical diagnosis, and follow-up	15,680
Pan et al. 2019 [20]	NA	NA	Histopathology	242
Yamakawa et al. 2019 [21]	NA	NA	NA	980
Hamm et al. 2019 [23]	Patients who (1) were untreated; (2) underwent locoregional therapy more than one year ago and now presented with a residual tumor	Patients younger than 18 years	Histopathology	296
Brehar et al. 2020 [26]	NA	NA	NA	268
Stollmaye et al. 2021 [35]	Patients who were either histologically confirmed or exhibited typical characteristics of the given lesion type with MRI	Patients younger than 18 years	NA	69
Kutlu et al. 2019 [19]	NA	NA	NA	345
Amita et al. 2019 [22]	NA	NA	NA	225
Zheng et al. 2021 [36]	Patients with (1) the presence of cirrhosis; (2) lesion size ≤ 2 cm; (3) <1-month interval between MRI and pathological examination	Patients whose (1) examinations had not been performed using Philips Ingenia equipment; (2) had a history of extrahepatic malignant tumors; (3) a history of local treatment for HCC; (4) severe motion artifacts detected between DCE-MRI and DWI (>5 slices of misalignment).	Histopathology, imaging features	120
Jia et al. 2019 [24]	Patients who were diagnosed as HCC by pathology examination	NA	Histopathology	99
Hassan et al. 2017 [49]	NA	NA	NA	110
Yasaka et al. 2017 [50]	Patients with five categories of liver masses or mass-like lesions (hereafter, we will refer to these as liver masses unless otherwise specified) of any size that were diagnosed based on the criteria described in the next subsection: HCCs; malignant liver tumors other than classic and early HCCs; indeterminate masses or mass-like lesions; liver hem-angiomas; cysts	Patients who (1) had CT image sets with prominent artifacts; (2) had those liver masses treated with transarterial chemoembolization therapy or systemic chemotherapy, and those liver masses; (3) were younger than 20 years	Histopathology	560
Bharti et al. 2018 [51]	NA	NA	NA	94
Schmauch et al.2019 [52]	NA	NA	NA	117
Mitrea et al. 2019 [53]	NA	NA	NA	300
Wang et al. 2020 [54]	NA	NA	Histopathology	235
Kim et al. 2021 [55]	Patients who (1) had chronic hepatitis B or liver cirrhosis; (2) underwent multiphase CT, consisting of late arterial, portal venous, and delayed phases; (3) underwent liver MRI within four months of CT scans; and (4) had available standard references, including pathologic evaluation or follow-up images	NA	Histopathology and follow-up	1086
Căleanu et al. 2021 [56]	NA	NA	NA	596
Chen et al. 2021 [57]	NA	NA	NA	NA
Chen et al. 2022 [58]	NA	patients with (1) a history of previous treatment such as surgery or interventional therapy; (2) diffuse liver disease such as diffuse cirrhosis, diffuse-type HCC, or diffuse metastatic tumor; (3) images with severe artifacts or incomplete scanning	Histopathology and follow-up	2189
Xiao et al. 2022 [59]	NA	NA	NA	135
Phan et al. 2023 [60]	NA	NA	NA	2000
Khan et al. 2023 [61]	NA	NA	Histopathology	68
Feng et al. 2023 [62]	NA	NA	NA	1241
Xu et al. 2023 [63]	NA	NA	NA	2333
Kim et al. 2023 [64]	NA	NA	NA	1062
Roy et al. 2023 [65]	NA	NA	NA	1080
Balasubramanian et al. 2023 [66]	NA	NA	NA	NA

Note. MF-ICC = mass-forming intrahepatic cholangiocarcinoma; ICC = Intrahepatic cholangiocellular carcinoma; HCC = hepatocellular carcinoma; HCH = hepatic cavernous hemangioma; FLLs = Focal liver lesions; FNH = focal nodular hyperplasia; DWI = diffusion-weighted imaging; HEM = cavernous hemangioma; HEP = hepatic abscess (HEP); MET = hepatic metastasis MET; NA = not available.

**Table 2:** Methods of model training and validation.

First Author and Year	Device	Exclusion of Poor Quality Imaging	Heatmap Provided	Methods Architecture	Type of Internal	External Validation	DL Versus Clinicians
					Validation		
Liu et al. 2023 [41]	MRI	NA	No	CNN-Oestmann, Inception v3, Densenet169, EfficientNet, VGG19, AlexNet, SFFNet	NA	No	No
Murtada et al. 2023 [42]	US	NO	No	ResNet152V2-559-Dense(128), densnet169-590-Dense(4096), densnet201-692-Dense(128)	cross-validation	No	No
Abhishek et al. 2023 [43]	CT	Yes	Yes	VGG, ResNet, DensNet, Inception v3, modified Inception v3	NA	No	Yes
Anisha et al.2023 [44]	CT	Yes	No	densenet201, InceptionResnetV2	NA	yes	No
Huang et al. 2023 [45]	CT	Yes	No	CSAM-Net, SE-Net	ten-fold cross-validation	No	No
Zhang et al. 2023 [47]	CT	Yes	No	MIDC-net	NA	No	No
Mitrea et al. 2023 [46]	US	No	No	ResNet101, InceptionV3, EfficientNet_b0, EfficientNet_ASPP, ConvNext_base	NA	No	No
Wang et al. 2023 [48]	CT	Yes	No	VGG16, VGG19, EI-CNNet, Inception V3, Xception, CNN	NA	No	No
Ling et al. 2022 [38]	CT	Yes	Yes	3D ResNet	five-fold cross-validation	No	Yes
Cao et al. 2022 [39]	CT	No	No	CNN	NA	No	No
Zhang et al. 2022 [40]	US	No	No	Xception, MobileNet, Resnet50, DenseNet121, InceptionV3	five-fold cross-validation	Yes	No
Gao et al. 2021 [30]	CT	Yes	No	CNN, RNN	five-fold cross-validation	Yes	Yes
Oestmann et al. 2021 [34]	MRI	Yes	No	CNN	Monte Carlo cross-validation	No	No
Wang et al. 2021 [31]	CT	Yes	Yes	HCCNet	NA	Yes	Yes
Wang et al. 2021 [33]	MRI	Yes	No	2D Densent121	five-fold cross-validation	No	No
Wang et al. 2021 [37]	MRI	Yes	Yes	3D ResNet-18	five-fold cross-validation	No	Yes
Zhou et al. 2021 [32]	US	Yes	Yes	Resnet 18	NA	No	No
Shi et al. 2020 [27]	CT	NA	No	MP-CDNs	NA	No	No
Zhen et al. 2020 [25]	MRI	Yes	Yes	Google Inception-ResNet V2	five-fold cross-validation	Yes	Yes
Kim et al. 2020 [28]	MRI	Yes	No	CNN	NA	Yes	Yes
Cao et al. 2020 [29]	CT	NA	Yes	MP-CDN	NA	No	No
Pan et al. 2019 [20]	US	NA	No	3D-CNN, DCCA-MKL	ten-fold cross-validation	No	No
Yamakawa et al. 2019 [21]	US	NA	No	VGGNet	cross-validation	No	No
Hamm et al. 2019 [23]	MRI	Yes	No	CNN	Monte Carlo cross-validation	No	Yes
Brehar et al. 2020 [26]	US	NA	No	VGGNet, ResNet, InceptionNet, DenseNet, SqueezeNet, Multi-Resolution CNN	NA	No	No
Stollmaye et al. 2021 [35]	MRI	NA	Yes	DenseNet264	five-fold cross-validation	No	No
Kutlu et al. 2019 [19]	CT	NA	No	CNN-DWT-LSTM	three-fold cross-validation	No	No
Amita et al. 2019 [22]	CT	NA	Yes	DNN	Monte Carlo cross-validation	No	Yes
Zheng et al. 2021 [36]	MRI	NA	No	PM-DL	NA	No	No
Jia et al. 2019 [24]	MRI	NA	Yes	ResNet	NA	No	No
Hassan et al. 2017 [49]	US	Yes	No	SSAE	ten-fold cross-validation	No	No
Yasaka et al. 2017 [50]	CT	No	No	CNN	NA	No	No
Bharti et al. 2018 [51]	US	No	No	CNN	cross-validation	No	No
Schmauch et al.2019 [52]	US	No	Yes	ResNet50	three-fold cross-validation	No	No
Mitrea et al. 2019 [53]	US	No	No	CNN	five-fold cross-validation	No	No
Wang et al. 2020 [54]	CT	No	Yes	SCCNN	NA	No	No
Kim et al. 2021 [55]	CT	Yes	Yes	R-CNN	NA	No	No
Căleanu et al. 2021 [56]	US	Yes	No	CNN	five-fold LOPO cross-validation	No	No
Chen et al. 2021 [57]	CT	NA	No	SED	NA	No	No
Chen et al. 2022 [58]	CT	NA	No	CNN	NA	No	No
Xiao et al. 2022 [59]	MRI	NA	No	CNN	five-fold cross-validation	No	No
Phan et al. 2023 [60]	CT	NA	No	R-CNN	cross-validation	No	No
Khan et al. 2023 [61]	CT	No	Yes	CNN	NA	No	No
Feng et al. 2023 [62]	US	No	No	Resnet50	five-fold cross-validation	No	No
Xu et al. 2023 [63]	CT	No	Yes	MCCNet	NA	No	No
Kim et al. 2023 [64]	US	No	No	3D-CNN, CNN-LSTM	ten/five-fold cross-validation	No	No
Roy et al. 2023 [65]	CT	Yes	No	CNN	ten-fold cross-validation	No	No
Balasubram-anian et al. 2023 [66]	CT	No	No	R-CNN	NA	No	No

Note-NA = Not available.

## Data Availability

We obtained the data from Embase, IEEE, PubMed, Web of Science, and the Cochrane Library databases.

## Supplementary Materials

The following supporting information can be downloaded at: https://www.mdpi.com/article/10.3390/cancers15235701/s1. Supplementary File S1: QUADAS-2 summary plot. Supplementary File S2: Publication bias and Meta-regression result. Supplementary File S3: Forest plot of studies included in the meta-analysis. Supplementary Note SI: The search terms and search strategy used in each database.

## References

[1] Sung H., Ferlay J., Siegel R.L., Laversanne M., Soerjomataram I., Jemal A., Bray F. (2021). Global Cancer Statistics 2020: GLOBOCAN Estimates of Incidence and Mortality Worldwide for 36 Cancers in 185 Countries. CA Cancer J. Clin..

[2] Vogel A., Meyer T., Sapisochin G., Salem R., Saborowski A. (2022). Hepatocellular carcinoma. Lancet.

[3] Lovet J.M., Kelley R.K., Villanueva A., Singal A.G., Pikarsky E., Roayaie S., Lencioni R., Koike K., Zucman-Rossi J., Finn R.S. (2021). Hepatocellular carcinoma. Nat. Rev. Dis. Primers.

[4] Zhang Q., Lou Y., Yang J., Wang J., Feng J., Zhao Y., Wang L., Huang X., Fu Q., Ye M. (2019). Integrated multi-omic analysis reveals comprehensive tumor heterogeneity and novel immunophenotypic classification in hepatocellular carcinomas. Gut.

[5] Yang J.D., Heimbach J.K. (2020). New advances in the diagnosis and management of hepatocellular carcinoma. BMJ.

[6] Kitao A., Matsui O., Zhang Y., Ogi T., Nakada S., Sato Y., Harada K., Yoneda N., Kozaka K., Inoue D. (2023). Dynamic CT and Gadoxetic Acid-enhanced MRI Characteristics of P53-mutated Hepatocellular Carcinoma. Radiology.

[7] Singal A.G., Lampertico P., Nahon P. (2020). Epidemiology and surveillance for hepatocellular carcinoma: New trends. J. Hepatol..

[8] Zhang S., Zhang J., Tian B., Lukasiewicz T., Xu Z. (2023). Multi-modal contrastive mutual learning and pseudo-label re-learning for semi-supervised medical image segmentation. Med. Image Anal..

[9] Kim D.H., Hong S.B., Choi S.H., Kim S.Y., Shim J.H., Lee J.S., Choi J.I., Kim S. (2022). Surveillance failure in ultrasound for hepatocellular carcinoma: A systematic review and meta-analysis. Gut.

[10] Chen R.J., Lu M.Y., Williamson D.F.K., Chen T.Y., Lipkova J., Noor Z., Shaban M., Shady M., Williams M., Joo B. (2022). Pan-cancer integrative histology-genomic analysis via multimodal deep learning. Cancer Cell.

[11] Bera K., Braman N., Gupta A., Velcheti V., Madabhushi A. (2022). Predicting cancer outcomes with radionics and artificial intelligence in radiology. Nat. Rev. Clin. Oncol..

[12] Kleppe A., Skrede O.J., De Raedt S., Hveem T.S., Askautrud H.A., Jacobsen J.E., Church D.N., Nesbakken A., Shepherd N.A., Novelli M. (2022). A clinical decision support system optimizing adjuvant chemotherapy for colorectal cancers by integrating deep learning and pathological staging markers: A development and validation study. Lancet Oncol..

[13] Hosny A., Parmar C., Quackenbush J., Schwartz L.H., Aerts H.J.W.L. (2018). Artificial intelligence in radiology. Nat. Rev. Cancer.

[14] Wang F., Casalino L.P., Khullar D. (2019). Deep Learning in Medicine-Promise, Progress, and Challenges. JAMA Intern. Med..

[15] Liu X., Rivera S.C., Moher D., Calvert M.J., Denniston A.K. (2020). SPIRIT-AI and CONSORT-AI Working Group. Reporting guidelines for clinical trial reports for interventions involving artificial intelligence: The CONSORT-AI Extension. BMJ.

[16] Page M.J., McKenzie J.E., Bossuyt P.M., Boutron I., Hoffmann T.C., Mulrow C.D., Shamseer L., Tetzlaff J.M., Akl E.A., Brennan S.E. (2021). The PRISMA 2020 statement: An updated guideline for reporting systematic reviews. Int. J. Surg..

[17] Shea B.J., Reeves B.C., Wells G., Thuku M., Hamel C., Moran J., Moher D., Tugwell P., Welch V., Kristjansson E. (2017). Amstar 2: A critical appraisal tool for systematic reviews that include randomized or non-randomized studies of healthcare interventions, or both. BMJ.

[18] Whiting P.F., Rutjes A.W., Westwood M.E., Mallett S., Deeks J.J., Reitsma J.B., Leeflang M.M., Sterne J.A., Bossuyt P.M., QUADAS-2 Group (2011). QUADAS-2: A revised tool for the quality assessment of diagnostic accuracy studies. Ann. Intern. Med..

[19] Kutlu H., Avcı E. (2019). A Novel Method for Classifying Liver and Brain Tumors Using Convolutional Neural Networks, Discrete Wavelet Transform and Long Short-Term Memory Networks. Sensors.

[20] Pan F., Huang Q., Li X. (2019). Classification of liver tumors with CEUS based on 3D-CNN. Proceedings of the 2019 IEEE 4th international conference on advanced robotics and mechatronics (ICARM).

[21] Yamakawa M., Shiina T., Nishida N. (2019). Computer-aided diagnosis system developed for ultrasound diagnosis of liver lesions using deep learning. Proceedings of the 2019 IEEE International Ultrasonics Symposium (IUS).

[22] Das A., Acharya U.R., Panda S.S. (2019). Deep learning based liver cancer detection using watershed transform and Gaussian mixture model techniques. Cogn. Syst. Res..

[23] Hamm C.A., Wang C.J., Savic L.J. (2019). Deep learning for liver tumor diagnosis part I: Development of a convolutional neural network classifier for multi-phasic MRI. Eur. Radiol..

[24] Jia X., Xiao Y., Yang D. (2019). Multi-parametric MRIs based assessment of hepatocellular carcinoma differentiation with multi-scale ResNet. KSII Trans. Internet Inf. Syst. TIIS.

[25] Zhen S.H., Cheng M., Tao Y.B., Wang Y.F., Juengpanich S., Jiang Z.Y., Jiang Y.K., Yan Y.Y., Lu W., Lue J.M. (2020). Deep Learning for Accurate Diagnosis of Liver Tumor Based on Magnetic Resonance Imaging and Clinical Data. Front. Oncol..

[26] Brehar R., Mitrea D.A., Vancea F., Marita T., Nedevschi S., Lupsor-Platon M., Rotaru M., Badea R.I. (2020). Comparison of Deep-Learning and Conventional Machine-Learning Methods for the Automatic Recognition of the Hepatocellular Carcinoma Areas from Ultrasound Images. Sensors.

[27] Shi W., Kuang S., Cao S., Hu B., Xie S., Chen S., Chen Y., Gao D., Chen Y., Zhu Y. (2020). Deep learning assisted differentiation of hepatocellular carcinoma from focal liver lesions: Choice of four-phase and three-phase CT imaging protocol. Abdom. Radiol..

[28] Kim J., Min J.H., Kim S.K., Shin S.Y., Lee M.W. (2020). Detection of Hepatocellular Carcinoma in Contrast-Enhanced Magnetic Resonance Imaging Using Deep Learning Classifier: A Multi-Center Retrospective Study. Sci. Rep..

[29] Cao S.E., Zhang L.Q., Kuang S.C., Shi W.Q., Hu B., Xie S.D., Chen Y.N., Liu H., Chen S.M., Jiang T. (2020). Multiphase convolutional dense network for the classification of focal liver lesions on dynamic contrast-enhanced computed tomography. World J. Gastroenterol..

[30] Gao R., Zhao S., Aishanjiang K., Cai H., Wei T., Zhang Y., Liu Z., Zhou J., Han B., Wang J. (2021). Deep learning for differential diagnosis of malignant hepatic tumors based on multi-phase contrast-enhanced CT and clinical data. J. Hematol. Oncol..

[31] Wang M., Fu F., Zheng B., Bai Y., Wu Q., Wu J., Sun L., Liu Q., Liu M., Yang Y. (2021). Development of an AI system for accurately diagnosing hepatocellular carcinoma from computed tomography imaging data. Br. J. Cancer.

[32] Zhou H., Jiang T., Li Q., Zhang C., Zhang C., Liu Y., Cao J., Sun Y., Jin P., Luo J. (2021). US-Based Deep Learning Model for Differentiating Hepatocellular Carcinoma (HCC) From Other Malignancy in Cirrhotic Patients. Front. Oncol..

[33] Wang P., Wu Y., Lai B. (2021). A deep learning pipeline for localization, differentiation, and uncertainty estimation of liver lesions using multi-phasic and multi-sequence MRI. arXiv.

[34] Oestmann P.M., Wang C.J., Savic L.J., Hamm C.A., Stark S., Schobert I., Gebauer B., Schlachter T., Lin M., Weinreb J.C. (2021). Deep learning-assisted differentiation of pathologically proven atypical and typical hepatocellular carcinoma (HCC) versus non-HCC on contrast-enhanced MRI of the liver. Eur. Radiol..

[35] Stollmayer R., Budai B.K., Tóth A., Kalina I., Hartmann E., Szoldán P., Bérczi V., Maurovich-Horvat P., Kaposi P.N. (2021). Diagnosis of focal liver lesions with deep learning-based multi-channel analysis of hepatocyte-specific contrast-enhanced magnetic resonance imaging. World J. Gastroenterol..

[36] Zheng R., Wang L., Wang C., Yu X., Chen W., Li Y., Li W., Yan F., Wang H., Li R. (2021). Feasibility of automatic detection of small hepatocellular carcinoma (≤2 cm) in cirrhotic liver based on pattern matching and deep learning. Phys. Med. Biol..

[37] Wang S.H., Han X.J., Du J., Wang Z.C., Yuan C., Chen Y., Zhu Y., Dou X., Xu X.W., Xu H. (2021). Saliency-based 3D convolutional neural network for categorizing common focal liver lesions on multisequence MRI. Insights Imaging.

[38] Ling Y., Ying S., Xu L., Peng Z., Mao X., Chen Z., Ni J., Liu Q., Gong S., Kong D. (2022). Automatic volumetric diagnosis of hepatocellular carcinoma based on four-phase CT scans with minimum extra information. Front. Oncol..

[39] Cao Y., Yu J., Zhang H., Xiong J., Luo Z. (2022). Classification of hepatic cavernous hemangioma or hepatocellular carcinoma using a convolutional neural network model. J. Gastrointest. Oncol..

[40] Zhang W.B., Hou S.Z., Chen Y.L., Mao F., Dong Y., Chen J.G., Wang W.P. (2022). Deep Learning for Approaching Hepatocellular Carcinoma Ultrasound Screening Dilemma: Identification of α-Fetoprotein-Negative Hepatocellular Carcinoma From Focal Liver Lesion Found in High-Risk Patients. Front. Oncol..

[41] Liu Y., Wang B., Mo X., Tang K., He J., Hao J. (2022). A Deep Learning Workflow for Mass-Forming Intrahepatic Cholangiocarcinoma and Hepatocellular Carcinoma Classification Based on MRI. Curr. Oncol..

[42] Elbashir M.K., Mahmoud A., Mostafa A.M. (2023). A Transfer Learning Approach Based on Ultrasound Images for Liver Cancer Detection. Comput. Mater. Contin..

[43] Midya A., Chakraborty J., Srouji R. (2023). Computerized Diagnosis of Liver Tumors from CT Scans Using a Deep Neural Network Approach. IEEE J. Biomed. Health Inform..

[44] Anisha A., Jiji G., Ajith B., Raj T. (2023). Deep feature fusion and optimized feature selection based ensemble classification of liver lesions. Imaging Sci. J..

[45] Huang J.L., Sun Y., Wu Z.H., Zhu H.J., Xia G.J., Zhu X.S., Wu J.H., Zhang K.H. (2023). Differential diagnosis of hepatocellular carcinoma and intrahepatic cholangiocarcinoma based on spatial and channel attention mechanisms. J. Cancer Res. Clin. Oncol..

[46] Mitrea D.A., Brehar R., Nedevschi S., Lupsor-Platon M., Socaciu M., Badea R. (2023). Hepatocellular Carcinoma Recognition from Ultrasound Images Using Combinations of Conventional and Deep Learning Techniques. Sensors.

[47] Zhang X., Jia N., Wang Y. (2023). Multi-input dense convolutional network for classification of hepatocellular carcinoma and intrahepatic cholangiocarcinoma. Biomed. Signal Process. Control.

[48] Wang X., Li N., Yin X., Xing L., Zheng Y. (2023). Classification of metastatic hepatic carcinoma and hepatocellular carcinoma lesions using contrast-enhanced CT based on EI-CNNet. Med. Phys..

[49] Hassan T.M., Elmogy M., Sallam E.S. (2017). Diagnosis of focal liver diseases based on deep learning technique for ultrasound images. Arab. J. Sci. Eng..

[50] Yasaka K., Akai H., Abe O., Kiryu S. (2018). Deep Learning with Convolutional Neural Network for Differentiation of Liver Masses at Dynamic Contrast-enhanced CT: A Preliminary Study. Radiology.

[51] Bharti P., Mittal D., Ananthasivan R. (2018). Preliminary Study of Chronic Liver Classification on Ultrasound Images Using an Ensemble Model. Ultrason. Imaging.

[52] Schmauch B., Herent P., Jehanno P., Dehaene O., Saillard C., Aubé C., Luciani A., Lassau N., Jégou S. (2019). Diagnosis of focal liver lesions from ultrasound using deep learning. Diagn. Interv. Imaging.

[53] Mitrea D., Nedevschi S., Mitrea P., Lupşor M.P., Badea R. (2019). HCC Recognition Within Ultrasound Images Employing Advanced Textural Features with Deep. Learning Techniques. Proceedings of the 2019 12th International Congress on Image and Signal Processing, BioMedical Engineering and Informatics (CISP-BMEI).

[54] Wang Q., Wang Z., Sun X., Zhang W., Li Y., Ge X., Huang Y., Liu Y., Chen Y. (2020). SCCNN: A diagnosis method for hepatocellular carcinoma and intrahepatic cholangiocarcinoma based on siamese cross contrast neural network. IEEE Access.

[55] Kim D.W., Lee G., Kim S.Y., Ahn G., Lee J.G., Lee S.S., Kim K.W., Park S.H., Lee Y.J., Kim N. (2021). Deep learning-based methods to detect primary hepatic malignancy in multiphase CT of patients at high risk for HCC. Eur. Radiol..

[56] Căleanu C.D., Sîrbu C.L., Simion G. (2021). Deep Neural Architectures for Contrast Enhanced Ultrasound (CEUS) Focal Liver Lesions Automated Diagnosis. Sensors.

[57] Chen W.F., Ou H.Y., Liu K.H., Li Z.Y., Liao C.C., Wang S.Y., Huang W., Cheng Y.F., Pan C.T. (2020). In-Series U-Net Network to 3D Tumor Image Reconstruction for Liver Hepatocellular Carcinoma Recognition. Diagnostics.

[58] Chen Q., Zhu Y., Chen Y., Wang F., Hu X., Ye Y., Dou X., Huang Y., Deng L., Zhou W. (2023). Applicability of multidimensional convolutional neural networks on automated detection of diverse focal liver lesions in multiphase CT images. Med. Phys..

[59] Xiao X., Zhao J., Li S. (2022). Task relevance driven adversarial learning for simultaneous detection, size grading, and quantification of hepatocellular carcinoma via integrating multi-modality MRI. Med. Image Anal..

[60] Phan A.C., Cao H.P., Le T.N., Trieu T.N., Phan T.C. (2023). Improving liver lesions classification on CT/MRI images based on Hounsfield Units attenuation and deep learning. Gene Expr. Patterns.

[61] Khan R.A., Fu M., Burbridge B., Luo Y., Wu F.X. (2023). A multi-modal deep neural network for multi-class liver cancer diagnosis. Neural Netw..

[62] Feng X., Cai W., Zheng R., Tang L., Zhou J., Wang H., Huang Q. (2023). Diagnosis of hepatocellular carcinoma using a deep network with multi-view enhanced patterns mined in contrast-enhanced ultrasound data. Eng. Appl. Artif. Intell..

[63] Xu X., Zhu Q., Ying H., Li J., Cai X., Li S., Liu X., Yu Y. (2023). A Knowledge-Guided Framework for Fine-Grained Classification of Liver Lesions Based on Multi-Phase CT Images. IEEE J. Biomed. Health Inform..

[64] Kim N., Lee W.J., Lee H.J. (2023). Deep learning classification of focal liver lesions with contrast-enhanced ultrasound from arterial phase recordings. Proceedings of the International Conference on Electronics, Information, and Communication (ICEIC).

[65] Roy S.S., Roy S., Mukherjee P., Roy S.S. (2023). An automated liver tumor segmentation and classification model by deep learning based approaches. Comput. Methods Biomech. Biomed. Eng. Imaging Vis..

[66] Balasubramanian P.K., Lai W.C., Seng G.H., Selvaraj J. (2023). APESTNet with Mask R-CNN for Liver Tumor Segmentation and Classification. Cancers.

[67] Chou R., Cuevas C., Fu R., Devine B., Wasson N., Ginsburg A., Sullivan S.D. (2015). Imaging Techniques for the Diagnosis of Hepatocellular Carcinoma: A Systematic Review and Meta-analysis. Ann. Intern. Med..

[68] Lai Q., Spoletini G., Mennini G., Laureiro Z.L., Tsilimigras D.I., Pawlik T.M., Rossi M. (2020). Prognostic role of artificial intelligence among patients with hepatocellular cancer: A systematic review. World J. Gastroenterol..

[69] Martinino A., Aloulou M., Chatterjee S., Scarano Pereira J.P., Singhal S., Patel T., Giovinazzo F. (2022). Artificial Intelligence in the Diagnosis of Hepatocellular Carcinoma: A Systematic Review. J. Clin. Med..

[70] Zhang J., Huang S., Xu Y., Wu J. (2022). Diagnostic Accuracy of Artificial Intelligence Based on Imaging Data for Preoperative Prediction of Microvascular Invasion in Hepatocellular Carcinoma: A Systematic Review and Meta-Analysis. Front. Oncol..

[71] Ravì D., Wong C., Deligianni F., Berthelot M., Andreu-Perez J., Lo B., Yang G.Z. (2017). Deep Learning for Health Informatics. IEEE J. Biomed. Health Inform..

[72] Zhou D., Tian F., Tian X., Sun L., Huang X., Zhao F., Li X. (2020). Diagnostic evaluation of a deep learning model for optical diagnosis of colorectal cancer. Nat. Commun..

[73] Xue P., Wang J., Qin D., Yan H., Qu Y., Seery S., Qiao Y. (2022). Deep learning in image-based breast and cervical cancer detection: A systematic review and meta-analysis. NPJ Digit. Med..

[74] Havaei M., Davy A., Warde-Farley D., Biard A., Courville A., Bengio Y., Larochelle H. (2017). Brain tumor segmentation with Deep Neural Networks. Med. Image Anal..

[75] Greenspan H., Van Ginneken B., Summers R.M. (2016). Guest editorial deep learning in medical imaging: Overview and future promise of an exciting new technique. IEEE Trans. Med. Imaging.

[76] Zhou L.Q., Wang J.Y., Yu S.Y., Wu G.G., Wei Q., Deng Y.B., Dietrich C.F. (2019). Artificial intelligence in medical imaging of the liver. World J. Gastroenterol..

[77] Wang C.J., Hamm C.A., Savic L.J., Ferrante M., Schobert I., Schlachter T., Letzen B. (2019). Deep learning for liver tumor diagnosis part II: Convolutional neural network interpretation using radiologic imaging features. Eur. Radiol..

[78] Esteva A., Kuprel B., Novoa R.A., Ko J., Swetter S.M., Blau H.M., Thrun S. (2017). Dermatologist-level classification of skin cancer with deep neural networks. Nature.

[79] Xu H.L., Gong T.T., Liu F.H., Chen H.Y., Xiao Q., Hou Y., Huang Y., Sun H.Z., Shi Y., Gao S. (2022). Artificial intelligence performance in image-based ovarian cancer identification: A systematic review and meta-analysis. EClinicalMedicine.

[80] Soffer S., Ben-Cohen A., Shimon O., Amitai M.M., Greenspan H., Klang E. (2019). Convolutional Neural Networks for Radiologic Images: A Radiologist’s Guide. Radiology.

