# Supplementary material for: Deep Learning Methods in Medical Image-Based Hepatocellular Carcinoma Diagnosis: A Systematic Review and Meta-Analysis
Source: Cancers (Basel). 2023 Dec 3;15(23):5701. doi: 10.3390/cancers15235701 (PMC10705136; doi:10.3390/cancers15235701)

Supplementary Online Conte

Deep Learning Algorithms in Medical Image-based Hepatocellular Carcinoma Diagnosis: A Systematic Review and Meta-analysis

Supplementary Figure S1: QUADAS-2 summary plot. Risk of bias and applicability concerns summary about each QUADAS-2 domain presented as percentages across the 48 included studies [19–66]

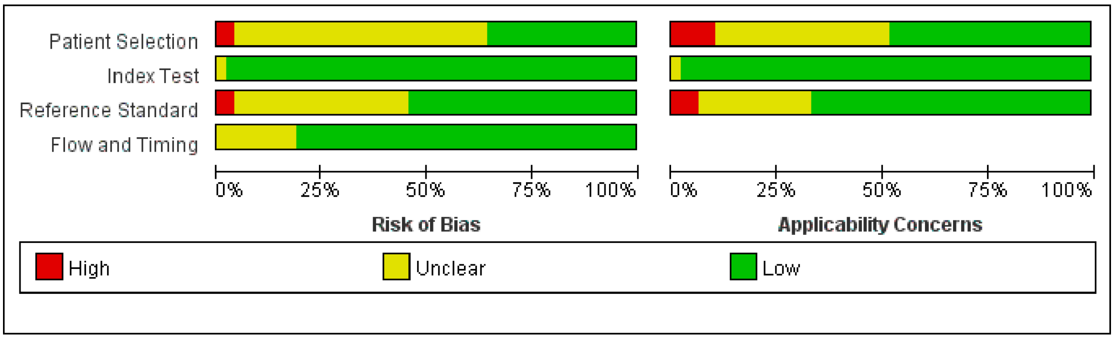

Supplementary Figure S2: Risk of bias and concern of applicability for each item in included studies [19–66].

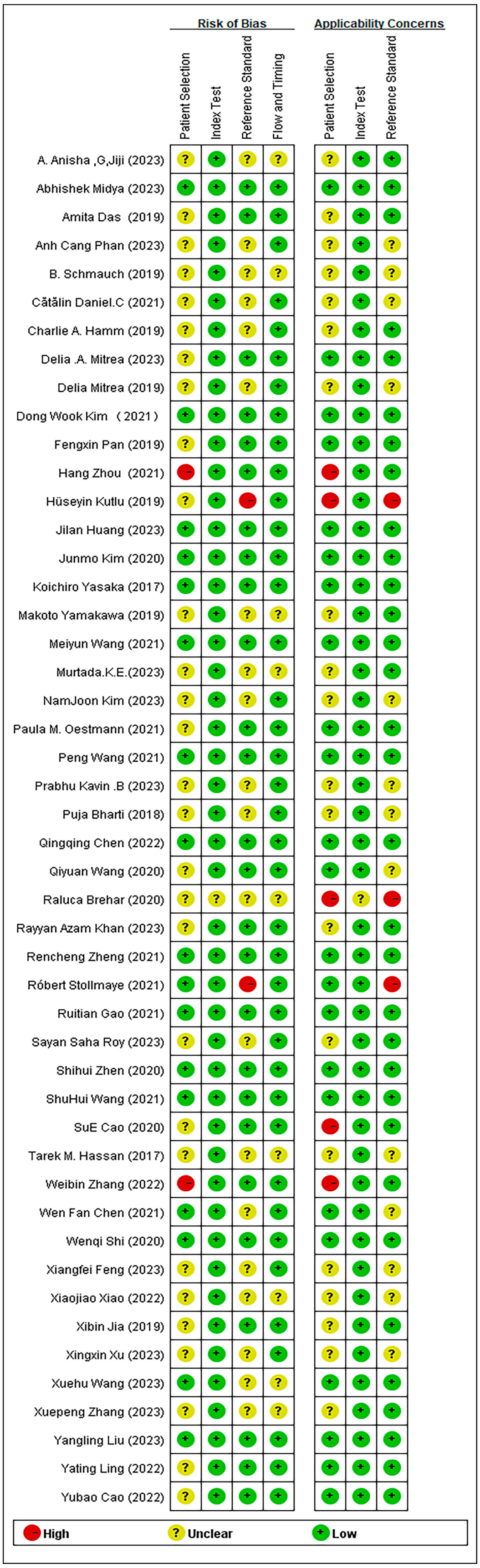

Supplement: Supplementary file 1 [file cancers-15-05701-s001.zip › Supplementary File S1.pdf]
