# Supplementary material for: Deep Learning Methods in Medical Image-Based Hepatocellular Carcinoma Diagnosis: A Systematic Review and Meta-Analysis
Source: Cancers (Basel). 2023 Dec 3;15(23):5701. doi: 10.3390/cancers15235701 (PMC10705136; doi:10.3390/cancers15235701)
Supplement: Supplementary file 1 [file cancers-15-05701-s001.zip › Supplementary File S2.pdf]

Supplementary Figure S3: Publication bias.

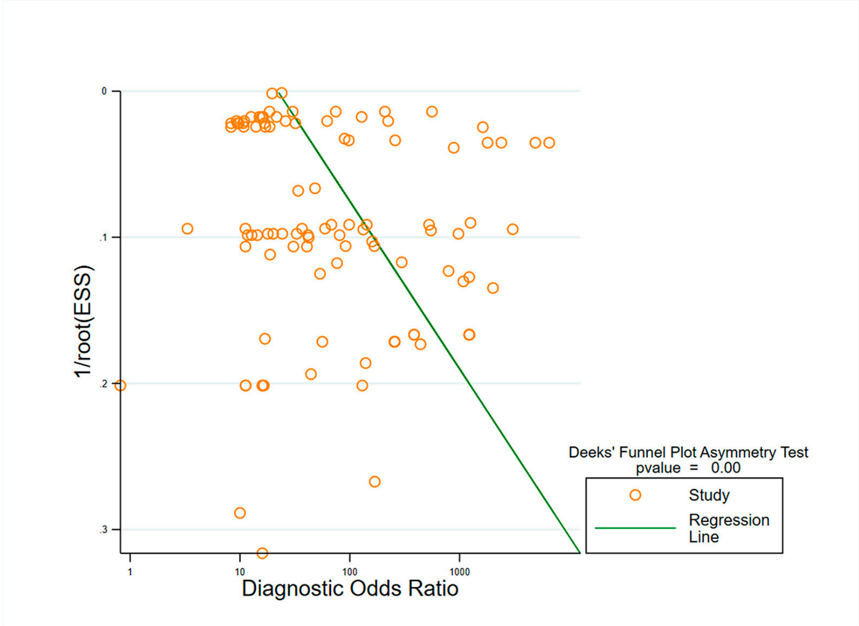

Supplementary Table S1: Meta-regression result.

Covariates	Subgroup	LRTChi2	I^2	*P* value
Image method	Contrast-enhanced images	11.20	82	<0.05
	Non-contrast-enhanced images			
Imaging modalities	MRI	0.25	0	0.88
	Ultrasound			
	CT			
Validation type	Internal validation	17.85	89	<0.05
	External validation			
