# Supplementary material for: Deep Learning Methods in Medical Image-Based Hepatocellular Carcinoma Diagnosis: A Systematic Review and Meta-Analysis
Source: Cancers (Basel). 2023 Dec 3;15(23):5701. doi: 10.3390/cancers15235701 (PMC10705136; doi:10.3390/cancers15235701)
Supplement: Supplementary file 1 [file cancers-15-05701-s001.zip › Supplementary File S3.pdf]

Supplementary Figure S4. Forest plot of studies included in the meta-analysis [19–48].

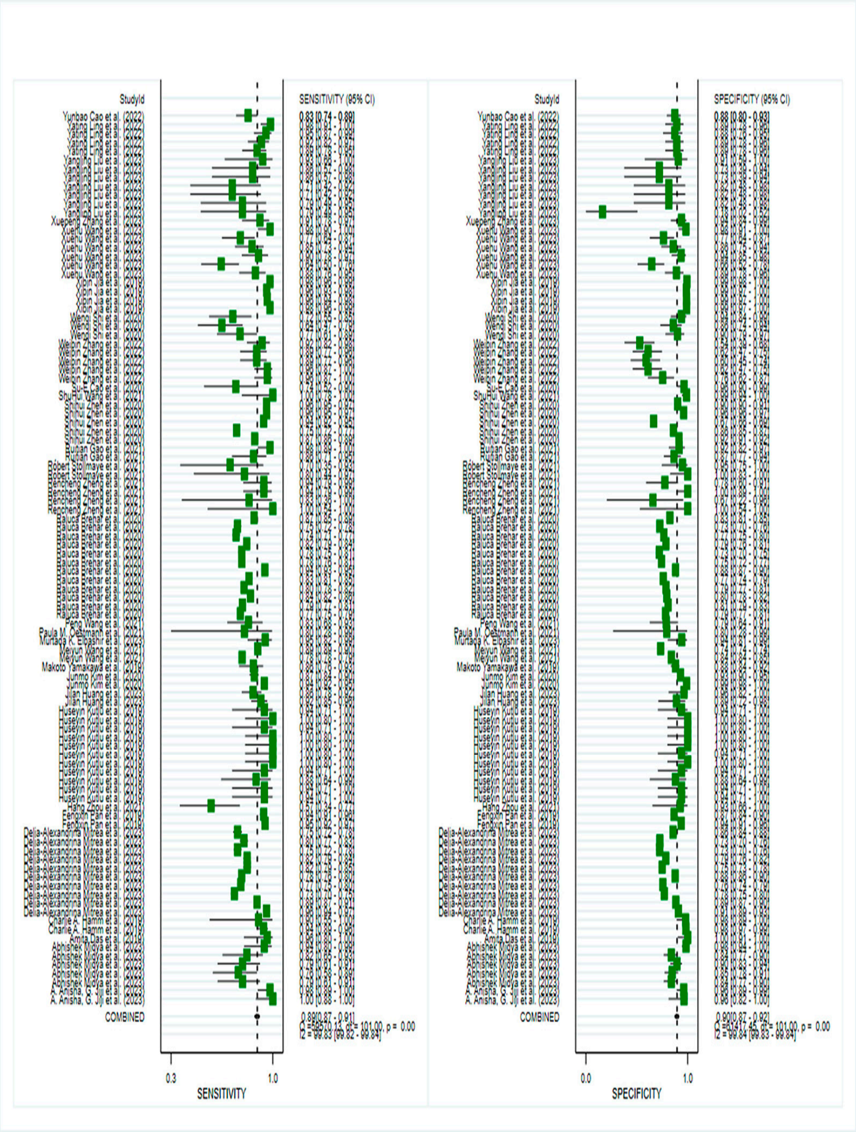

Supplementary Figure S5: Forest plot of different medical image features (contrast-enhanced [19,20,22–25,27–38,43–45,47,48] and non-contrast-enhanced images [21,26,39,40,42,46]).

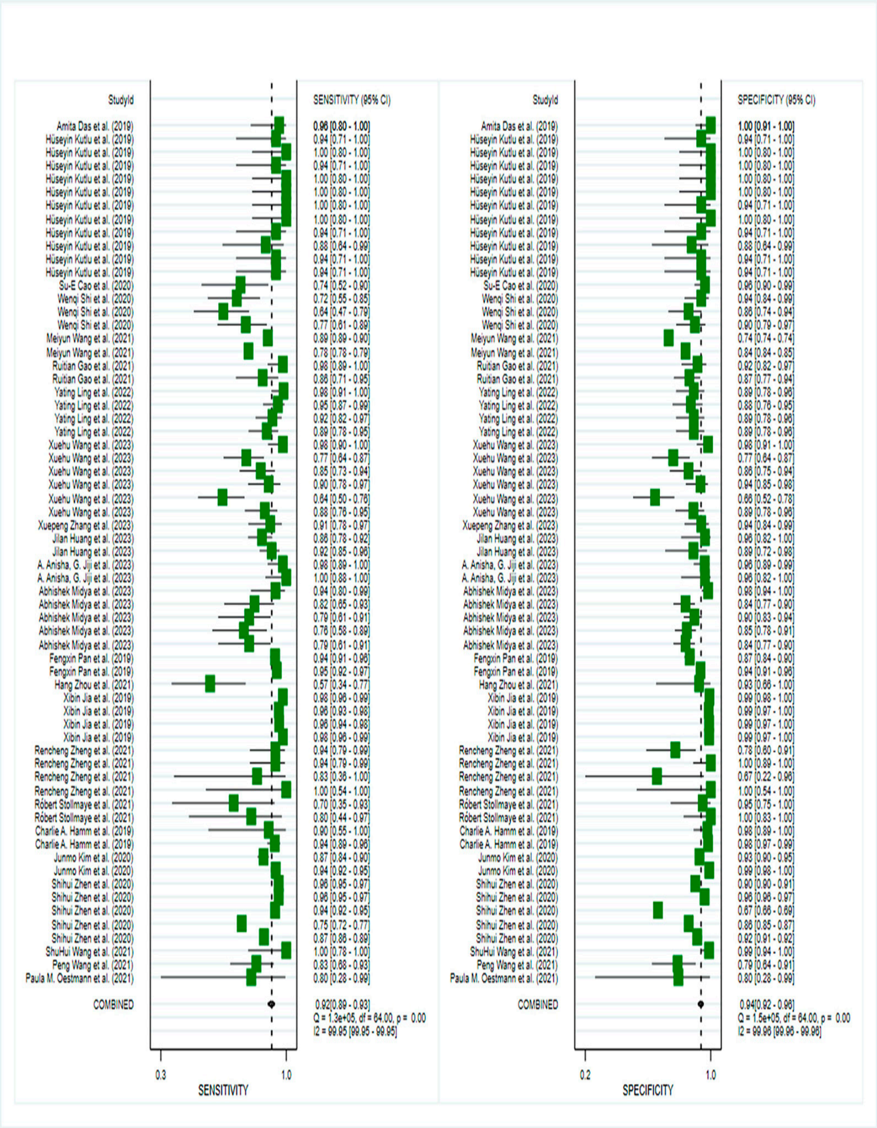

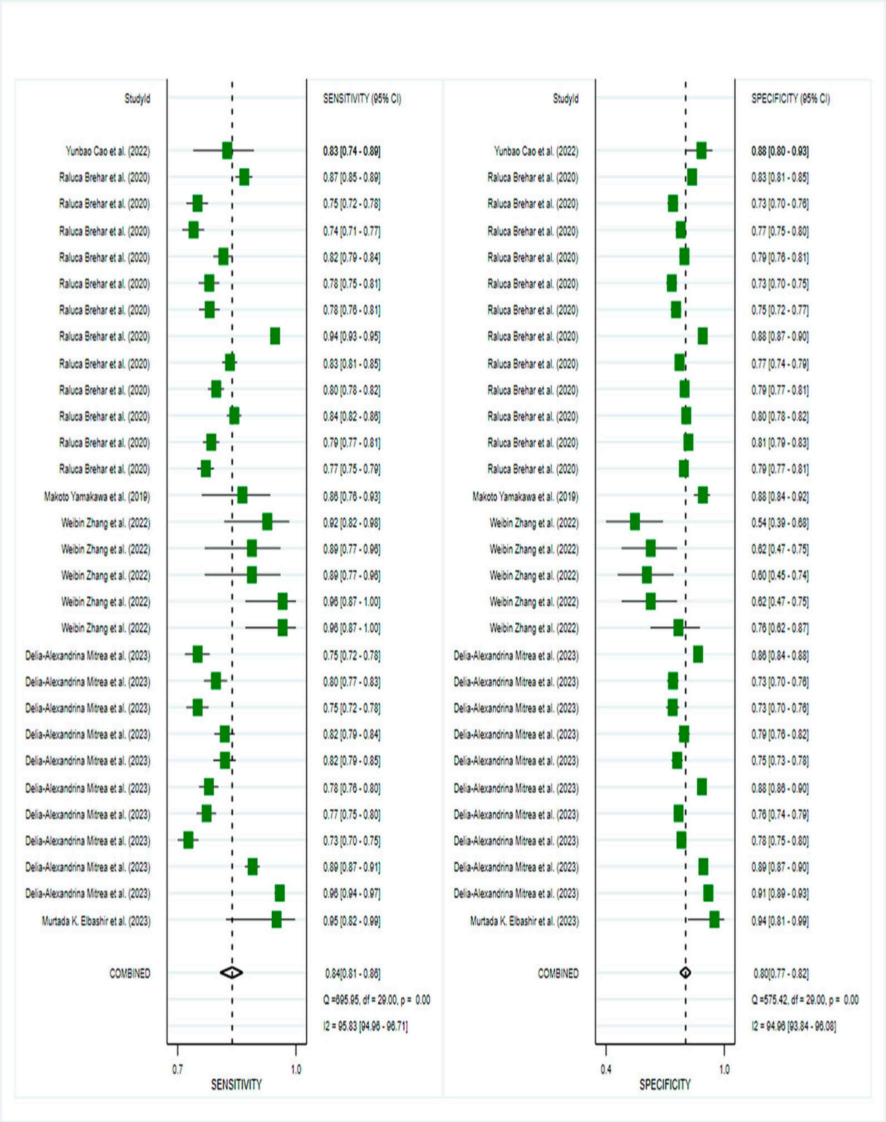
StudyId
SENSITIVITY (95% CI)
Yunbao Cao et al. (2022)
0.83 [0.74 - 0.89]
Raluca Brehar et al. (2020)
0.87 [0.85 - 0.89]
Raluca Brehar et al. (2020)
0.75 [0.72 - 0.78]
Raluca Brehar et al. (2020)
0.74 [0.71 - 0.77]
Raluca Brehar et al. (2020)
0.82 [0.79 - 0.84]
Raluca Brehar et al. (2020)
0.78 [0.75 - 0.81]
Raluca Brehar et al. (2020)
0.78 [0.76 - 0.81]
Raluca Brehar et al. (2020)
0.94 [0.93 - 0.95]
Raluca Brehar et al. (2020)
0.83 [0.81 - 0.85]
Raluca Brehar et al. (2020)
0.80 [0.78 - 0.82]
Raluca Brehar et al. (2020)
0.84 [0.82 - 0.86]
Raluca Brehar et al. (2020)
0.79 [0.77 - 0.81]
Raluca Brehar et al. (2020)
0.77 [0.75 - 0.79]
Makoto Yamakawa et al. (2019)
0.86 [0.76 - 0.93]
Weibin Zhang et al. (2022)
0.92 [0.82 - 0.98]
Weibin Zhang et al. (2022)
0.89 [0.77 - 0.96]
Weibin Zhang et al. (2022)
0.89 [0.77 - 0.96]
Weibin Zhang et al. (2022)
0.96 [0.87 - 1.00]
Weibin Zhang et al. (2022)
0.96 [0.87 - 1.00]
Delia-Alexandrina Mitrea et al. (2023)
0.75 [0.72 - 0.78]
Delia-Alexandrina Mitrea et al. (2023)
0.80 [0.77 - 0.83]
Delia-Alexandrina Mitrea et al. (2023)
0.75 [0.72 - 0.78]
Delia-Alexandrina Mitrea et al. (2023)
0.82 [0.79 - 0.84]
Delia-Alexandrina Mitrea et al. (2023)
0.82 [0.79 - 0.85]
Delia-Alexandrina Mitrea et al. (2023)
0.78 [0.76 - 0.80]
Delia-Alexandrina Mitrea et al. (2023)
0.77 [0.75 - 0.80]
Delia-Alexandrina Mitrea et al. (2023)
0.73 [0.70 - 0.75]
Delia-Alexandrina Mitrea et al. (2023)
0.89 [0.87 - 0.91]
Delia-Alexandrina Mitrea et al. (2023)
0.96 [0.94 - 0.97]
Murtada K. Elbashir et al. (2023)
0.95 [0.82 - 0.99]
COMBINED
0.84[0.81 - 0.86]
Q =695.95, df = 29.00, p = 0.00
I2 = 95.83 [94.96 - 96.71]
0.7
1.0
SENSITIVITY
StudyId
SPECIFICITY (95% CI)
Yunbao Cao et al. (2022)
0.88 [0.80 - 0.93]
Raluca Brehar et al. (2020)
0.83 [0.81 - 0.85]
Raluca Brehar et al. (2020)
0.73 [0.70 - 0.76]
Raluca Brehar et al. (2020)
0.77 [0.75 - 0.80]
Raluca Brehar et al. (2020)
0.79 [0.76 - 0.81]
Raluca Brehar et al. (2020)
0.73 [0.70 - 0.75]
Raluca Brehar et al. (2020)
0.75 [0.72 - 0.77]
Raluca Brehar et al. (2020)
0.88 [0.87 - 0.90]
Raluca Brehar et al. (2020)
0.77 [0.74 - 0.79]
Raluca Brehar et al. (2020)
0.79 [0.77 - 0.81]
Raluca Brehar et al. (2020)
0.80 [0.78 - 0.82]
Raluca Brehar et al. (2020)
0.81 [0.79 - 0.83]
Raluca Brehar et al. (2020)
0.79 [0.77 - 0.81]
Makoto Yamakawa et al. (2019)
0.88 [0.84 - 0.92]
Weibin Zhang et al. (2022)
0.54 [0.39 - 0.68]
Weibin Zhang et al. (2022)
0.62 [0.47 - 0.75]
Weibin Zhang et al. (2022)
0.60 [0.45 - 0.74]
Weibin Zhang et al. (2022)
0.62 [0.47 - 0.75]
Weibin Zhang et al. (2022)
0.76 [0.62 - 0.87]
Delia-Alexandrina Mitrea et al. (2023)
0.86 [0.84 - 0.88]
Delia-Alexandrina Mitrea et al. (2023)
0.73 [0.70 - 0.76]
Delia-Alexandrina Mitrea et al. (2023)
0.73 [0.70 - 0.76]
Delia-Alexandrina Mitrea et al. (2023)
0.79 [0.76 - 0.82]
Delia-Alexandrina Mitrea et al. (2023)
0.75 [0.73 - 0.78]
Delia-Alexandrina Mitrea et al. (2023)
0.88 [0.86 - 0.90]
Delia-Alexandrina Mitrea et al. (2023)
0.76 [0.74 - 0.79]
Delia-Alexandrina Mitrea et al. (2023)
0.78 [0.75 - 0.80]
Delia-Alexandrina Mitrea et al. (2023)
0.89 [0.87 - 0.90]
Delia-Alexandrina Mitrea et al. (2023)
0.91 [0.89 - 0.93]
Murtada K. Elbashir et al. (2023)
0.94 [0.81 - 0.99]
COMBINED
0.80[0.77 - 0.82]
Q =575.42, df = 29.00, p = 0.00
I2 = 94.96 [93.84 - 96.08]
0.4
1.0
SPECIFICITY

Supplementary Figure S6: Forest plot of different imaging modalities (MRI [23–25,28,34,33,37,35,36,41], US [20,21,26,32,40,42,46], and CT [19,22,27,29,30,31,38,39,43–45,47,48,]).

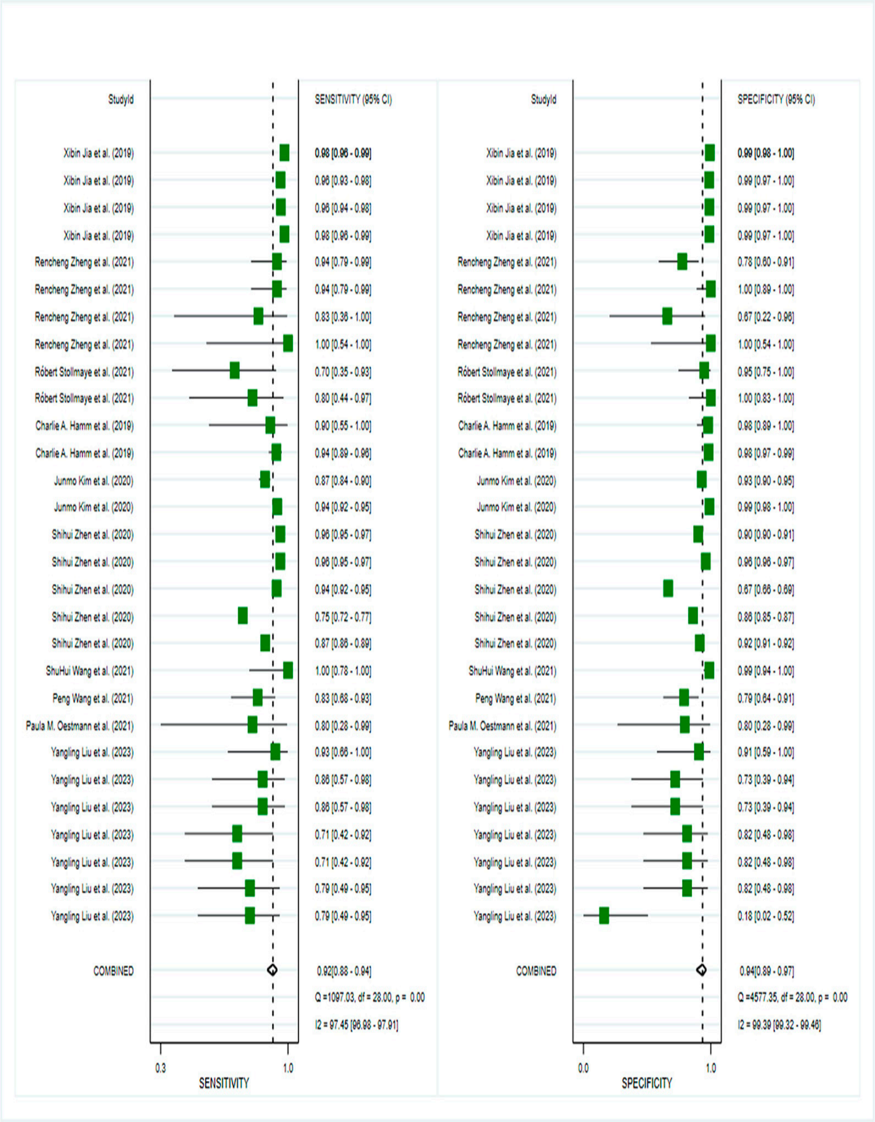

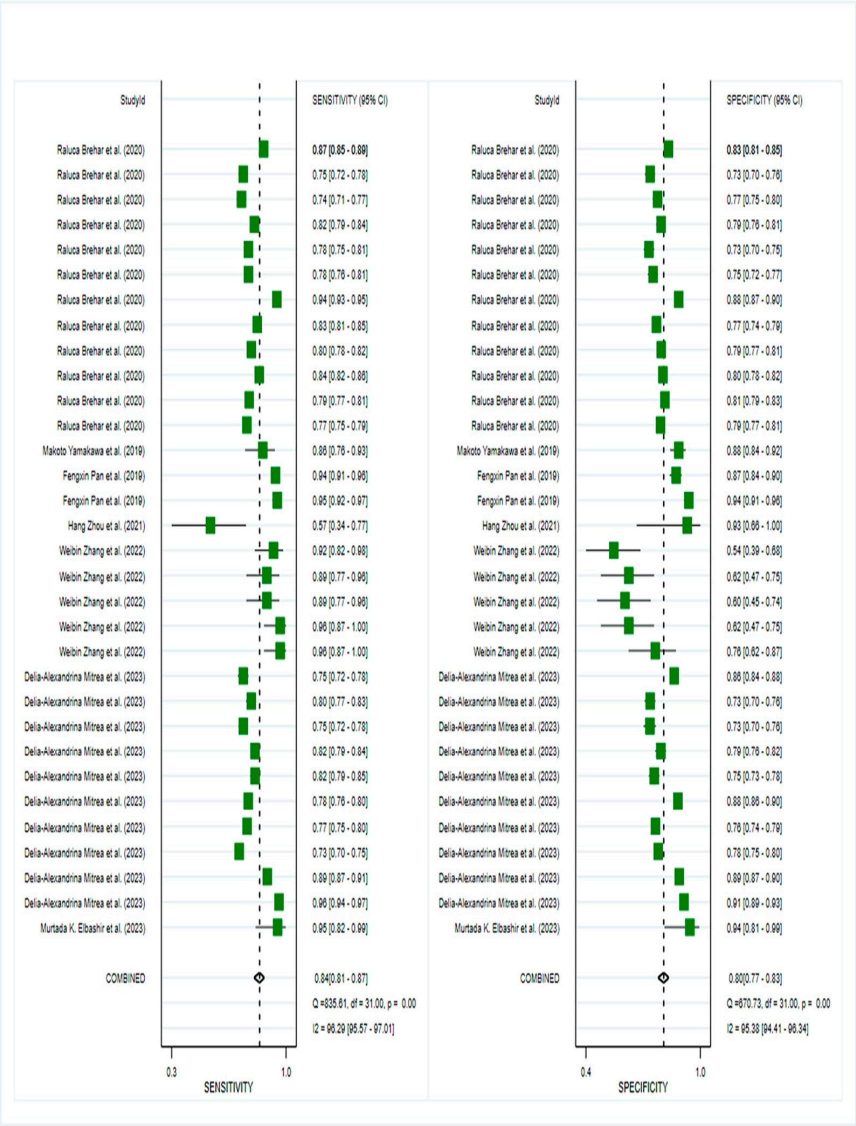
StudyId
SENSITIVITY (95% CI)
Raluca Brehar et al. (2020) 0.87 [0.85 - 0.89]
Raluca Brehar et al. (2020) 0.75 [0.72 - 0.78]
Raluca Brehar et al. (2020) 0.74 [0.71 - 0.77]
Raluca Brehar et al. (2020) 0.82 [0.79 - 0.84]
Raluca Brehar et al. (2020) 0.78 [0.75 - 0.81]
Raluca Brehar et al. (2020) 0.78 [0.76 - 0.81]
Raluca Brehar et al. (2020) 0.94 [0.93 - 0.95]
Raluca Brehar et al. (2020) 0.83 [0.81 - 0.85]
Raluca Brehar et al. (2020) 0.80 [0.78 - 0.82]
Raluca Brehar et al. (2020) 0.84 [0.82 - 0.86]
Raluca Brehar et al. (2020) 0.79 [0.77 - 0.81]
Raluca Brehar et al. (2020) 0.77 [0.75 - 0.79]
Makoto Yamakawa et al. (2019) 0.86 [0.76 - 0.93]
Fengxin Pan et al. (2019) 0.94 [0.91 - 0.96]
Fengxin Pan et al. (2019) 0.95 [0.92 - 0.97]
Hang Zhou et al. (2021) 0.57 [0.34 - 0.77]
Weibin Zhang et al. (2022) 0.92 [0.82 - 0.98]
Weibin Zhang et al. (2022) 0.89 [0.77 - 0.96]
Weibin Zhang et al. (2022) 0.89 [0.77 - 0.96]
Weibin Zhang et al. (2022) 0.96 [0.87 - 1.00]
Weibin Zhang et al. (2022) 0.96 [0.87 - 1.00]
Delia-Alexandrina Mitrea et al. (2023) 0.75 [0.72 - 0.78]
Delia-Alexandrina Mitrea et al. (2023) 0.80 [0.77 - 0.83]
Delia-Alexandrina Mitrea et al. (2023) 0.75 [0.72 - 0.78]
Delia-Alexandrina Mitrea et al. (2023) 0.82 [0.79 - 0.84]
Delia-Alexandrina Mitrea et al. (2023) 0.82 [0.79 - 0.85]
Delia-Alexandrina Mitrea et al. (2023) 0.78 [0.76 - 0.80]
Delia-Alexandrina Mitrea et al. (2023) 0.77 [0.75 - 0.80]
Delia-Alexandrina Mitrea et al. (2023) 0.73 [0.70 - 0.75]
Delia-Alexandrina Mitrea et al. (2023) 0.89 [0.87 - 0.91]
Delia-Alexandrina Mitrea et al. (2023) 0.96 [0.94 - 0.97]
Murtada K. Elbashir et al. (2023) 0.95 [0.82 - 0.99]
COMBINED 0.84[0.81 - 0.87]
Q =835.61, df = 31.00, p = 0.00
I2 = 96.29 [95.57 - 97.01]
0.3
1.0
SENSITIVITY
StudyId
SPECIFICITY (95% CI)
Raluca Brehar et al. (2020) 0.83 [0.81 - 0.85]
Raluca Brehar et al. (2020) 0.73 [0.70 - 0.76]
Raluca Brehar et al. (2020) 0.77 [0.75 - 0.80]
Raluca Brehar et al. (2020) 0.79 [0.76 - 0.81]
Raluca Brehar et al. (2020) 0.73 [0.70 - 0.75]
Raluca Brehar et al. (2020) 0.75 [0.72 - 0.77]
Raluca Brehar et al. (2020) 0.88 [0.87 - 0.90]
Raluca Brehar et al. (2020) 0.77 [0.74 - 0.79]
Raluca Brehar et al. (2020) 0.79 [0.77 - 0.81]
Raluca Brehar et al. (2020) 0.80 [0.78 - 0.82]
Raluca Brehar et al. (2020) 0.81 [0.79 - 0.83]
Raluca Brehar et al. (2020) 0.79 [0.77 - 0.81]
Makoto Yamakawa et al. (2019) 0.88 [0.84 - 0.92]
Fengxin Pan et al. (2019) 0.87 [0.84 - 0.90]
Fengxin Pan et al. (2019) 0.94 [0.91 - 0.96]
Hang Zhou et al. (2021) 0.93 [0.66 - 1.00]
Weibin Zhang et al. (2022) 0.54 [0.39 - 0.68]
Weibin Zhang et al. (2022) 0.62 [0.47 - 0.75]
Weibin Zhang et al. (2022) 0.60 [0.45 - 0.74]
Weibin Zhang et al. (2022) 0.62 [0.47 - 0.75]
Weibin Zhang et al. (2022) 0.76 [0.62 - 0.87]
Delia-Alexandrina Mitrea et al. (2023) 0.86 [0.84 - 0.88]
Delia-Alexandrina Mitrea et al. (2023) 0.73 [0.70 - 0.76]
Delia-Alexandrina Mitrea et al. (2023) 0.73 [0.70 - 0.76]
Delia-Alexandrina Mitrea et al. (2023) 0.79 [0.76 - 0.82]
Delia-Alexandrina Mitrea et al. (2023) 0.75 [0.73 - 0.78]
Delia-Alexandrina Mitrea et al. (2023) 0.88 [0.86 - 0.90]
Delia-Alexandrina Mitrea et al. (2023) 0.76 [0.74 - 0.79]
Delia-Alexandrina Mitrea et al. (2023) 0.78 [0.75 - 0.80]
Delia-Alexandrina Mitrea et al. (2023) 0.89 [0.87 - 0.90]
Delia-Alexandrina Mitrea et al. (2023) 0.91 [0.89 - 0.93]
Murtada K. Elbashir et al. (2023) 0.94 [0.81 - 0.99]
COMBINED 0.80[0.77 - 0.83]
Q =670.73, df = 31.00, p = 0.00
I2 = 95.38 [94.41 - 96.34]
0.4
1.0
SPECIFICITY

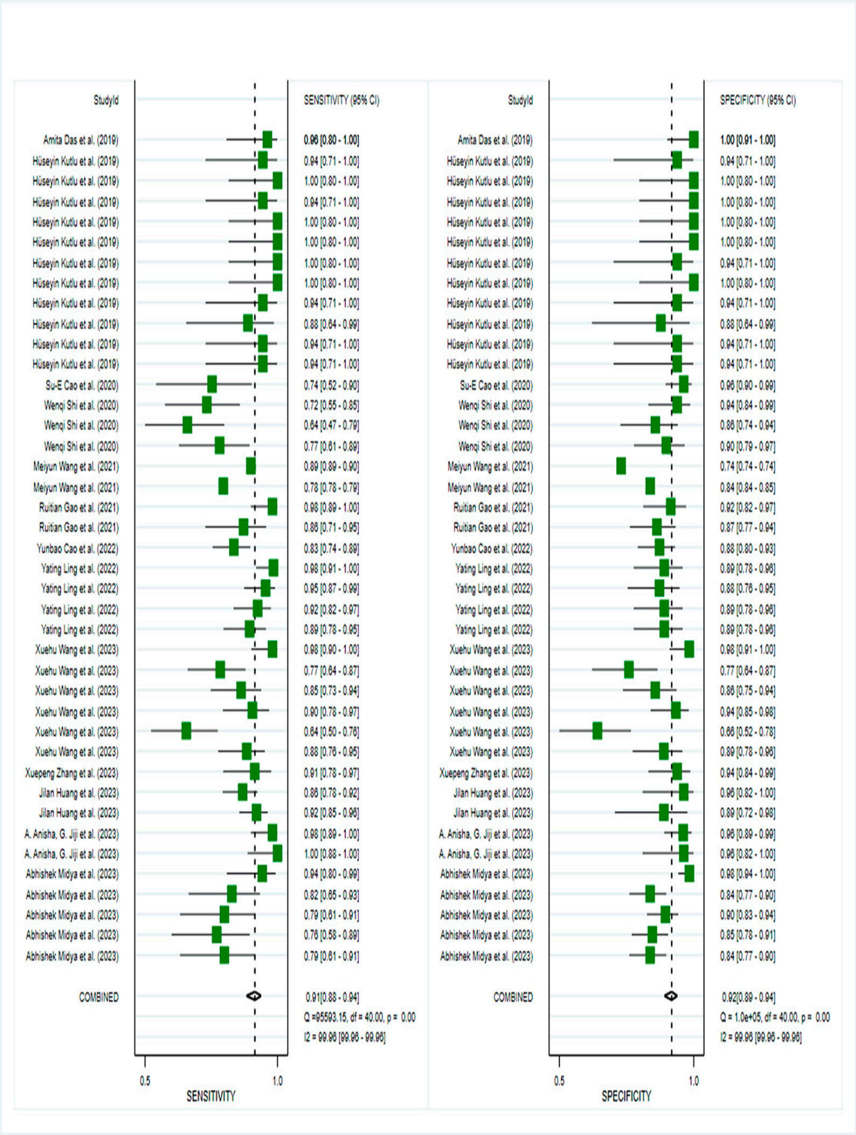
StudyId
SENSITIVITY (95% CI)
Amita Das et al. (2019) 0.96 [0.80 - 1.00]
Hüseyin Kutlu et al. (2019) 0.94 [0.71 - 1.00]
Hüseyin Kutlu et al. (2019) 1.00 [0.80 - 1.00]
Hüseyin Kutlu et al. (2019) 0.94 [0.71 - 1.00]
Hüseyin Kutlu et al. (2019) 1.00 [0.80 - 1.00]
Hüseyin Kutlu et al. (2019) 1.00 [0.80 - 1.00]
Hüseyin Kutlu et al. (2019) 1.00 [0.80 - 1.00]
Hüseyin Kutlu et al. (2019) 1.00 [0.80 - 1.00]
Hüseyin Kutlu et al. (2019) 0.94 [0.71 - 1.00]
Hüseyin Kutlu et al. (2019) 0.88 [0.64 - 0.99]
Hüseyin Kutlu et al. (2019) 0.94 [0.71 - 1.00]
Hüseyin Kutlu et al. (2019) 0.94 [0.71 - 1.00]
Su-E Cao et al. (2020) 0.74 [0.52 - 0.90]
Wenqi Shi et al. (2020) 0.72 [0.55 - 0.85]
Wenqi Shi et al. (2020) 0.64 [0.47 - 0.79]
Wenqi Shi et al. (2020) 0.77 [0.61 - 0.89]
Meiyun Wang et al. (2021) 0.89 [0.89 - 0.90]
Meiyun Wang et al. (2021) 0.78 [0.78 - 0.79]
Ruitian Gao et al. (2021) 0.98 [0.89 - 1.00]
Ruitian Gao et al. (2021) 0.86 [0.71 - 0.95]
Yunbao Cao et al. (2022) 0.83 [0.74 - 0.89]
Yating Ling et al. (2022) 0.98 [0.91 - 1.00]
Yating Ling et al. (2022) 0.95 [0.87 - 0.99]
Yating Ling et al. (2022) 0.92 [0.82 - 0.97]
Yating Ling et al. (2022) 0.89 [0.78 - 0.95]
Xuehu Wang et al. (2023) 0.98 [0.90 - 1.00]
Xuehu Wang et al. (2023) 0.77 [0.64 - 0.87]
Xuehu Wang et al. (2023) 0.85 [0.73 - 0.94]
Xuehu Wang et al. (2023) 0.90 [0.78 - 0.97]
Xuehu Wang et al. (2023) 0.64 [0.50 - 0.76]
Xuehu Wang et al. (2023) 0.88 [0.76 - 0.95]
Xuepeng Zhang et al. (2023) 0.91 [0.78 - 0.97]
Jilan Huang et al. (2023) 0.86 [0.78 - 0.92]
Jilan Huang et al. (2023) 0.92 [0.85 - 0.96]
A. Anisha, G. Jiji et al. (2023) 0.98 [0.89 - 1.00]
A. Anisha, G. Jiji et al. (2023) 1.00 [0.88 - 1.00]
Abhishek Midya et al. (2023) 0.94 [0.80 - 0.99]
Abhishek Midya et al. (2023) 0.82 [0.65 - 0.93]
Abhishek Midya et al. (2023) 0.79 [0.61 - 0.91]
Abhishek Midya et al. (2023) 0.76 [0.58 - 0.89]
Abhishek Midya et al. (2023) 0.79 [0.61 - 0.91]
COMBINED 0.91[0.88 - 0.94]
Q =95593.15, df = 40.00, p = 0.00
I2 = 99.96 [99.96 - 99.96]
0.5
1.0
SENSITIVITY
StudyId
SPECIFICITY (95% CI)
Amita Das et al. (2019) 1.00 [0.91 - 1.00]
Hüseyin Kutlu et al. (2019) 0.94 [0.71 - 1.00]
Hüseyin Kutlu et al. (2019) 1.00 [0.80 - 1.00]
Hüseyin Kutlu et al. (2019) 1.00 [0.80 - 1.00]
Hüseyin Kutlu et al. (2019) 1.00 [0.80 - 1.00]
Hüseyin Kutlu et al. (2019) 1.00 [0.80 - 1.00]
Hüseyin Kutlu et al. (2019) 0.94 [0.71 - 1.00]
Hüseyin Kutlu et al. (2019) 1.00 [0.80 - 1.00]
Hüseyin Kutlu et al. (2019) 0.94 [0.71 - 1.00]
Hüseyin Kutlu et al. (2019) 0.88 [0.64 - 0.99]
Hüseyin Kutlu et al. (2019) 0.94 [0.71 - 1.00]
Hüseyin Kutlu et al. (2019) 0.94 [0.71 - 1.00]
Su-E Cao et al. (2020) 0.96 [0.90 - 0.99]
Wenqi Shi et al. (2020) 0.94 [0.84 - 0.99]
Wenqi Shi et al. (2020) 0.86 [0.74 - 0.94]
Wenqi Shi et al. (2020) 0.90 [0.79 - 0.97]
Meiyun Wang et al. (2021) 0.74 [0.74 - 0.74]
Meiyun Wang et al. (2021) 0.84 [0.84 - 0.85]
Ruitian Gao et al. (2021) 0.92 [0.82 - 0.97]
Ruitian Gao et al. (2021) 0.87 [0.77 - 0.94]
Yunbao Cao et al. (2022) 0.88 [0.80 - 0.93]
Yating Ling et al. (2022) 0.89 [0.78 - 0.96]
Yating Ling et al. (2022) 0.88 [0.76 - 0.95]
Yating Ling et al. (2022) 0.89 [0.78 - 0.96]
Yating Ling et al. (2022) 0.89 [0.78 - 0.96]
Xuehu Wang et al. (2023) 0.98 [0.91 - 1.00]
Xuehu Wang et al. (2023) 0.77 [0.64 - 0.87]
Xuehu Wang et al. (2023) 0.86 [0.75 - 0.94]
Xuehu Wang et al. (2023) 0.94 [0.85 - 0.98]
Xuehu Wang et al. (2023) 0.66 [0.52 - 0.78]
Xuehu Wang et al. (2023) 0.89 [0.78 - 0.96]
Xuepeng Zhang et al. (2023) 0.94 [0.84 - 0.99]
Jilan Huang et al. (2023) 0.96 [0.82 - 1.00]
Jilan Huang et al. (2023) 0.89 [0.72 - 0.98]
A. Anisha, G. Jiji et al. (2023) 0.96 [0.89 - 0.99]
A. Anisha, G. Jiji et al. (2023) 0.96 [0.82 - 1.00]
Abhishek Midya et al. (2023) 0.98 [0.94 - 1.00]
Abhishek Midya et al. (2023) 0.84 [0.77 - 0.90]
Abhishek Midya et al. (2023) 0.90 [0.83 - 0.94]
Abhishek Midya et al. (2023) 0.85 [0.78 - 0.91]
Abhishek Midya et al. (2023) 0.84 [0.77 - 0.90]
COMBINED 0.92[0.89 - 0.94]
Q = 1.0e+05, df = 40.00, p = 0.00
I2 = 99.96 [99.96 - 99.96]
0.5
1.0
SPECIFICITY

Supplementary Figure S7: Forest plot of different validation types (internal [19–39,41–48] or external [23,28,30,31,40]).

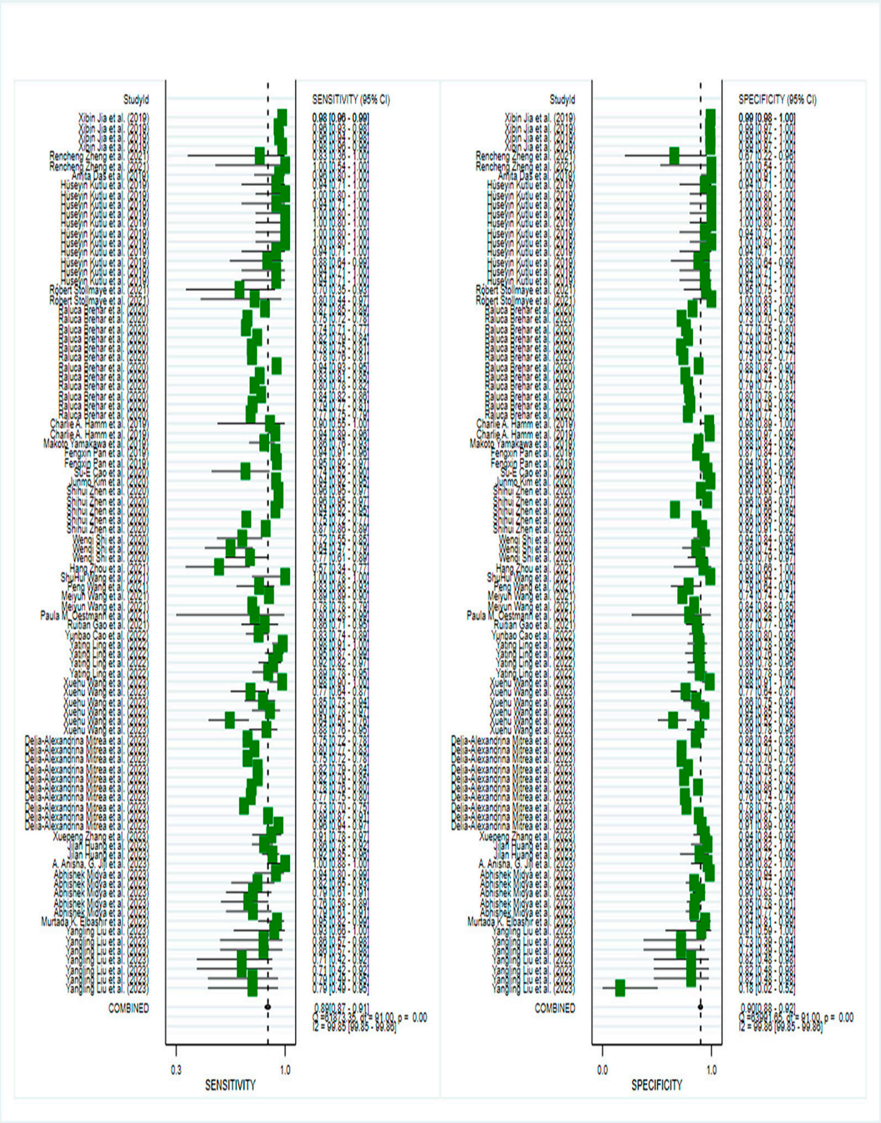

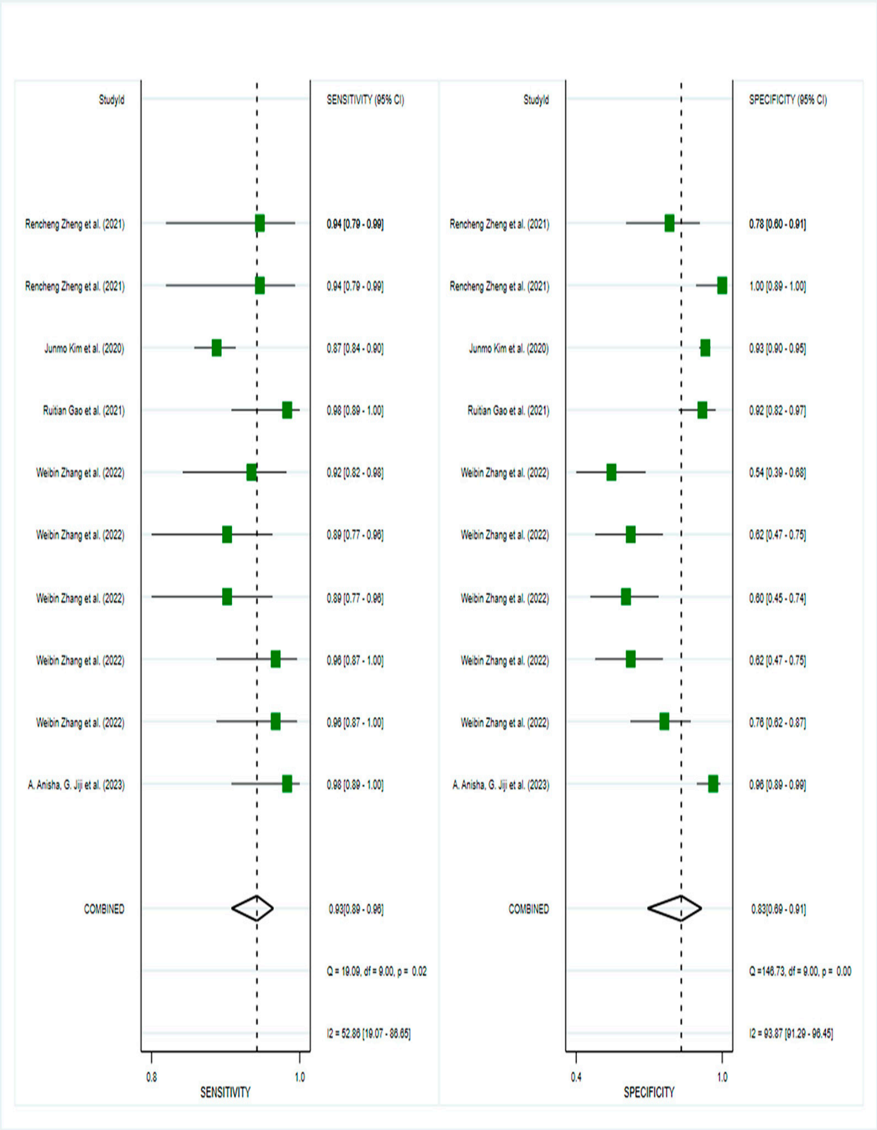
StudyId
SENSITIVITY (95% CI)
Rencheng Zheng et al. (2021)
0.94 [0.79 - 0.99]
Rencheng Zheng et al. (2021)
0.94 [0.79 - 0.99]
Junmo Kim et al. (2020)
0.87 [0.84 - 0.90]
Ruitian Gao et al. (2021)
0.98 [0.89 - 1.00]
Weibin Zhang et al. (2022)
0.92 [0.82 - 0.98]
Weibin Zhang et al. (2022)
0.89 [0.77 - 0.96]
Weibin Zhang et al. (2022)
0.89 [0.77 - 0.96]
Weibin Zhang et al. (2022)
0.96 [0.87 - 1.00]
Weibin Zhang et al. (2022)
0.96 [0.87 - 1.00]
A. Anisha, G. Jiji et al. (2023)
0.98 [0.89 - 1.00]
COMBINED
0.93[0.89 - 0.96]
Q = 19.09, df = 9.00, p = 0.02
I2 = 52.86 [19.07 - 86.65]
0.8
1.0
SENSITIVITY
StudyId
SPECIFICITY (95% CI)
Rencheng Zheng et al. (2021)
0.78 [0.60 - 0.91]
Rencheng Zheng et al. (2021)
1.00 [0.89 - 1.00]
Junmo Kim et al. (2020)
0.93 [0.90 - 0.95]
Ruitian Gao et al. (2021)
0.92 [0.82 - 0.97]
Weibin Zhang et al. (2022)
0.54 [0.39 - 0.68]
Weibin Zhang et al. (2022)
0.62 [0.47 - 0.75]
Weibin Zhang et al. (2022)
0.60 [0.45 - 0.74]
Weibin Zhang et al. (2022)
0.62 [0.47 - 0.75]
Weibin Zhang et al. (2022)
0.76 [0.62 - 0.87]
A. Anisha, G. Jiji et al. (2023)
0.96 [0.89 - 0.99]
COMBINED
0.83[0.69 - 0.91]
Q =146.73, df = 9.00, p = 0.00
I2 = 93.87 [91.29 - 96.45]
0.4
1.0
SPECIFICITY
