# Supplementary material for: Deep Learning Methods in Medical Image-Based Hepatocellular Carcinoma Diagnosis: A Systematic Review and Meta-Analysis
Source: Cancers (Basel). 2023 Dec 3;15(23):5701. doi: 10.3390/cancers15235701 (PMC10705136; doi:10.3390/cancers15235701)
Supplement: Supplementary file 1 [file cancers-15-05701-s001.zip › Supplementary Note SI. Search terms and search strategy .pdf]

Supplementary Note SI. Search terms and search strategy

Pubmed

Search: ((((Carcinoma, Hepatocellular[MeSH Terms]) OR (hepatocellular carcinoma[Title/Abstract])) OR (HCC[Title/Abstract])) AND (((Artificial intelligence[MeSH Terms]) OR (deep learning[Title/Abstract])) OR (neural network[Title/Abstract]))) AND ((((diagnosis[Title/Abstract]) OR (screen[Title/Abstract])) OR (classify[Title/Abstract])) OR (discriminate[Title/Abstract]))

Embase

#1 'artificial intelligence'/exp OR 'deep learning'/exp OR 'artificial neural network'/exp

#2 'diagnosis'/exp OR 'diagnostic procedure'/exp OR 'screening'/exp OR 'classification'/exp OR 'discriminate'

#3 'performance'/exp OR 'sensitivity and specificity'/exp OR 'diagnostic accuracy'/exp OR 'area under the curve'/exp

#4 'carcinoma, hepatocellular'/exp OR 'hepatocellular carcinoma'/exp OR 'HCC'

#5 #1 AND #2 AND #3 AND #4

Cochrane

("Artificial intelligence" OR "deep learning " OR neural network") AND ("diagnosis" OR "screening" OR " classification " OR " discriminate ") AND ("performance" OR "sensitivity" OR "specificity" OR "accuracy" OR "area under the curve" OR "auc" OR "calibrate ") AND ("Carcinoma, Hepatocellular" OR "hepatocellular carcinoma" OR "HCC")

Web of Science

((TS=(Artificial intelligence)) OR TS=(deep learning)) OR TS=(neural network)

(((TS=(diagnosis)) OR TS=(screening)) OR TS=(classification)) OR TS=(discriminate)

((((((TS=(performance)) OR TS=(sensitivity)) OR TS=(specificity)) OR TS=(accuracy)) OR TS=(area under the curve)) OR TS=(auc)) OR TS=(calibrate)

((TS=(Carcinoma, Hepatocellular)) OR TS=(hepatocellular carcinoma)) OR TS=(HCC)

IEEE

("All Metadata":"artificial intelligence" OR "All Metadata":"deep learning" OR "All Metadata":"neural network") AND ("All Metadata":"performance" OR "All Metadata":"sensitivity and specificity" OR "All Metadata":"accuracy" OR "All Metadata":"area under the curve") AND ("All Metadata":"screening" OR "All Metadata":"diagnosis" OR "All Metadata":"classification" OR "All Metadata":"discriminat") AND ("All Metadata":" Carcinoma, Hepatocellular " OR "All Metadata":"hepatocellular carcinoma "OR "All Metadata":"HCC")
